# Identification of proteins involved in the anti-inflammatory properties of *Propionibacterium freudenreichii* by means of a multi-strain study

**DOI:** 10.1038/srep46409

**Published:** 2017-04-13

**Authors:** Stéphanie-Marie Deutsch, Mahendra Mariadassou, Pierre Nicolas, Sandrine Parayre, Rozenn Le Guellec, Victoria Chuat, Vincent Peton, Caroline Le Maréchal, Julien Burati, Valentin Loux, Valérie Briard-Bion, Julien Jardin, Coline Plé, Benoît Foligné, Gwénaël Jan, Hélène Falentin

**Affiliations:** 1 STLO, UMR 1253, INRA, Agrocampus Ouest, 35000, Rennes, France; 2 MaIAGE, UR1404, INRA, 78352 Jouy-en-Josas, France; 3 Univ. Lille, CNRS, Inserm, CHU Lille, Institut Pasteur de Lille, U1019 – UMR 8204 - CIIL - Center for Infection and Immunity of Lille, 59000 Lille, France

## Abstract

*Propionibacterium freudenreichii*, a dairy starter, can reach a population of almost 10^9^ propionibacteria per gram in Swiss-type cheese at the time of consumption. Also consumed as a probiotic, it displays strain-dependent anti-inflammatory properties mediated by surface proteins that induce IL-10 in leukocytes. We selected 23 strains with varied anti-inflammatory potentials in order to identify the protein(s) involved. After comparative genomic analysis, 12 of these strains were further analysed by surface proteomics, eight of them being further submitted to transcriptomics. The omics data were then correlated to the anti-inflammatory potential evaluated by IL-10 induction. This comparative omics strategy highlighted candidate genes that were further subjected to gene-inactivation validation. This validation confirmed the contribution of surface proteins, including SlpB and SlpE, two proteins with SLH domains known to mediate non-covalent anchorage to the cell-wall. Interestingly, HsdM3, predicted as cytoplasmic and involved in DNA modification, was shown to contribute to anti-inflammatory activity. Finally, we demonstrated that a single protein cannot explain the anti-inflammatory properties of a strain. These properties therefore result from different combinations of surface and cytoplasmic proteins, depending on the strain. Our enhanced understanding of the molecular bases for immunomodulation will enable the relevant screening for bacterial resources with anti-inflammatory properties.

*Propionibacterium freudenreichii (Pf*) is a food grade bacterium with GRAS (generally recognized as safe) status that belongs to the dairy propionibacteria class. It is most commonly used as a starter for the manufacture of fermented dairy products such Emmental and Leerdammer cheeses^1^, but it is also employed as a probiotic supplement^2^. During the past decade, growing interest in pathologies linked to dysbiosis, such as inflammatory bowel disease, has motivated research on the anti-inflammatory properties of beneficial bacteria, including probiotic bacteria as *Pf*. The remarkable anti-inflammatory properties of the *Pf* species were revealed, but the genetic basis for them still needs to be elucidated. The first genome of the type strain CIRM 1 was published in 2010^3^. Since then, 22 genomes have been sequenced, annotated and made publicly available^4^,^5^,^6^. Interestingly, the *Pf* species is characterised by high intraspecific variability among strains regarding numerous physiological traits, including their immunomodulation properties; although the data on this latter trait have highlighted the remarkable anti-inflammatory properties of the species, their levels are strain-dependant^7^. *In vitro*, the probiotic JS strain of *Pf* was shown to inhibit the secretion of IL-8 from *Helicobacter pylori*-infected Caco-2 cells^8^. In a human peripheral blood mononuclear cell (PBMC) model, this strain stimulated IL-10 production up to levels similar to those measured for the three bifidobacteria strains tested^9^. In human PBMCs, nine strains of *Pf* were shown to induce the anti-inflammatory cytokine IL-10 with varying intensity, whereas no pro-inflammatory IL-12, TNF-α or IFN-γ cytokines were induced^7^. Within the same nine strains, SI48 was shown to protect mice from TNBS-induced colitis by modulating both colonic and systemic inflammatory markers. The *Pf* CIRM 129 strain (also known as ITG P20) was shown to induce IL-10 after growth on laboratory YEL culture medium and also on skimmed milk. Interestingly, when grown in combination with other lactic acid bacteria in fermented milk, this strain retained its anti-inflammatory properties and also reduced the pro-inflammatory IL-12 cytokines induced by lactic acid bacteria in the PBMC model^10^. The consumption by mice of an experimental cheese fermented with the same CIRM 129 strain alleviated the symptoms of TNBS-induced colitis^11^. In the same way, a milk whey culture of the *Pf* ET-3 strain administrated to rats with TNBS-colitis helped to accelerate the healing of colitis^12^. Finally, the consumption of milk fermented with the *Pf* CIRM 129 strain was shown to modulate cytokines in the colon of pigs^13^. The molecular mechanisms and genetic basis for the anti-inflammatory effects of *Pf* are still unclear. The involvement of chemical compounds secreted by *Pf* outside the cell has been suggested by different authors. In the study by Uchida & Mogami^12^, the healing of TNBS-induced colitis was also observed following the oral administration of propionate, the major metabolite released by Propionibacterium species. In mice with DSS-induced colitis, DHNA (1,4-Dihydroxy-2-naphthoic acid), which is a component of the menaquinone (vitamin K2) biosynthesis pathways produced by *Pf*, improved the survival rate and histological damage scores^14^. DHNA attenuates inflammation in interleukin-10-deficient mice with colitis by inhibiting the production of macrophage-derived pro-inflammatory cytokines^15^. The surface composition of *Pf* bacteria has been studied, sometimes in correlation with their immunomodulatory properties. Some strains are covered with a layer of β-glucan exopolysaccharides, whose synthesis is encoded by the *gtfF* gene^16^,^17^. In three *Pf* strains (CIRM1, lsp110 and lsp103), the *gtfF* gene was inactivated. This inactivation leads to a lack of β-glucan at the bacterial surface. Interestingly, the resulting mutants induce the release of cytokines, while the parental wild types do not, suggesting the availability of key surface components^18^. In three *Pf* strains (CIRM129, 118 and SI48), surface proteins have been shown to be involved in their anti-inflammatory properties. Three guanidine-treated strains lost their ability to induce IL-10, indicating that surface extractable proteins trigger the release of IL-10 immunomodulatory cytokine^7^,^19^. Identification of the proteins extracted by guanidine in CIRM129 revealed a mix of at least five proteins: SlpA, SlpB, SlpE, InlA and LspA^19^. The identity of the proteins responsible for the immune response is still unknown.

During the present study, we therefore hypothesised that the surface composition of *Pf* was linked to its immunomodulatory properties. To test this hypothesis, the surface proteome of *Pf* was characterized for 12 sequenced strains using three different, previously described methods^19^. We then investigated possible correlations between, on the one hand, the ability to induce IL-10 cytokine, and on the other, the presence of genes, the expression of mRNAs and detection in the surface proteome. For the first time, a multi-strain study combining comparative genomics, transcriptomics and surface proteomics, coupled with gene inactivation, led to the identification of key surface proteins responsible for the anti-inflammatory properties of *Pf* strains.

## Results

### Anti-inflammatory properties of P. freudenreichii strains

**Figure 1:**
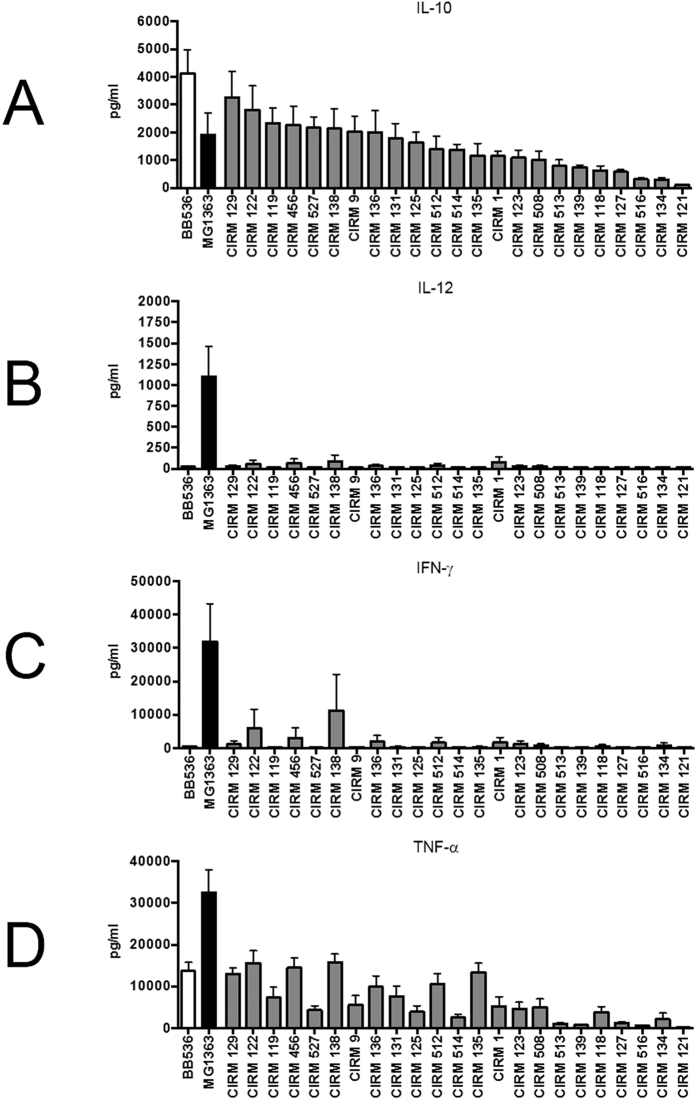
*In vitro* immunomodulatory phenotypes of *Propionibacterium freudenreichii* strains grown on dairy-based culture medium. Comparative anti-inflammatory (**A**) IL-10 and pro-inflammatory (**B**) IL-12, (**C**) IFN-γ and (**D**) TNF-α cytokine responses of human PBMCs stimulated by *Pf* strains or by the positive controls *Lactococcus lactis* MG1363 and *Bifidobacterium longum* BB536 (pro- and anti-inflammatory strains, respectively). All bacterial strains were implemented at a bacterial density corresponding to a multiplicity of infection (MOI) of 10, and the supernatants collected were analysed using ELISA. Immunocompetent PBMC cells were stimulated by bacteria for 24 h and the data are expressed in pg/ml as means +/− SEM of the results concerning four separate french healthy blood donors. The *Pf* strains are ordered by their decreasing induction of IL-10, which is highly variable.

Twenty-three strains of *Pf* were tested for their immunomodulatory properties, based on different cytokine induction patterns following the stimulation of human PBMCs, i.e. IL-10, IL-12, TNF-α and IFN-γ, and after growth in a dairy-based medium. A strain-dependent induction of the anti-inflammatory cytokine IL-10 was observed (Fig. 1). Some strains, such as CIRM 129 and CIRM 122, induced a high level of IL-10 secretion in PBMCs, similar to that induced by the anti-inflammatory BB536 strain of *Bifidobacterium longum*, used as positive control. By contrast, some strains such as CIRM 121 induced very low IL-10 secretion in PBMCs. The secretion of pro-inflammatory cytokines (IL-12, IFN-γ and TNF-α) by PBMCs was also analysed. As for *B. longum* BB536, *Pf* strains were seen to be very weak inducers of IFN-γ (except for CIRM 122 and CIRM 138) and did not induce IL-12. *Pf* CIRM 122 and *Pf* CIRM 138 induced IFN-γ secretion by PBMCs but to a lesser extent than that induced by MG1363, the weak anti-inflammatory strain of *Lactococcus lactis*. As for IL-10 secretion, *Pf*-induced TNF-α secretion was strain-dependent but the levels did not exceed those induced by the BB536 strain of *B. longum*. Collectively, these results tended to indicate an anti-inflammatory profile for all *Pf* strains, with varying levels of IL-10, weak levels of IFN-γ and TNF-α and no induction of IL-12.

### Phylogenetic analysis of P. freudenreichii strains and the selection of strains for association analysis

**Figure 2:**
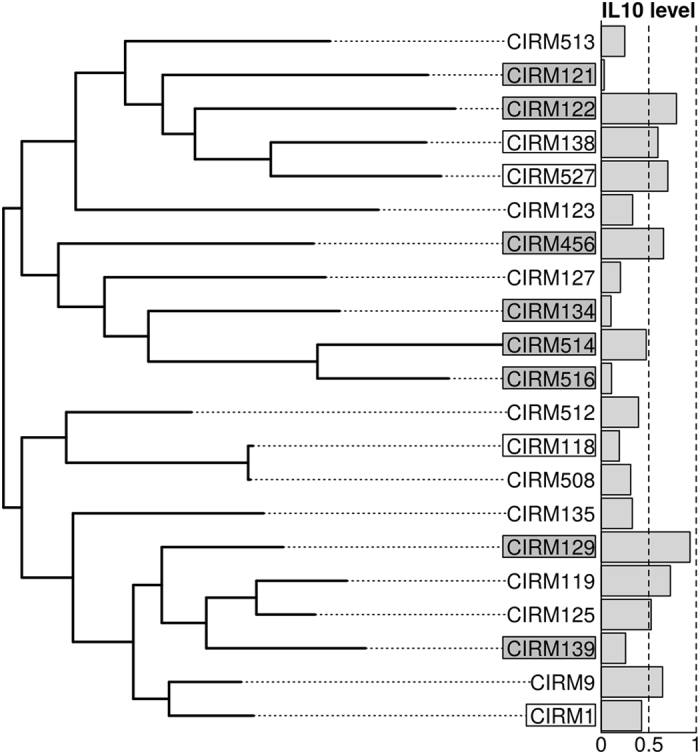
Maximum likelihood phylogenetic tree (JTT + I + G) of the *Propionibacterium freudenreichii* strains used in this study. The 12 strains shown in boxes were used in the proteomics study and the eight strains with a grey background were also used in the transcriptomics study. IL-10 levels were normalised within each donor using *B. longum* (BB536) induced levels as a reference and averaged over four donors.

The strains used for the phylogenetic analysis are shown in Supplementary Table S1 available online. The genome of CIRM 1, previously sequenced and annotated, is complete, whereas the other strains are in a draft state and deposited in several scaffolds in the EMBL database. In order to evaluate strain diversity, a phylogenetic tree was reconstructed, based on the concatenation of orthologous proteins in the core genome, *i*.e. proteins present in a single copy in all strains (1011 out of 4971 proteins) (Fig. 2). Twelve strains were preselected in pairs from this phylogenetic tree for additional proteomic studies. The strains were chosen in pairs so that: (i) members of a pair would be close by in the tree (genetically similar) but exhibit contrasted phenotypic properties, and (ii) the pairs would span the complete diversity of the phylogenetic tree, so as to mitigate the effect of the shared evolutionary history of strains (see Fig. 2). The 12 selected strains (boxed in Fig. 2) consisted of CIRM 1, 118, 121, 122, 129, 134, 138, 139, 456, 514, 516 and 527. Eight of them (shown in grey in Fig. 2, all previous strains excluding CIRM 1, 118, 138, 527) were also selected for transcriptomic studies: they included CIRM 129, 122 and 456 as strong anti-inflammatory strains, CIRM 516, 134 and 121 as weak anti-inflammatory strains and CIRM 514 and 139 as intermediate effect strains.

#### Surface proteome analysis of *P. freudenreichii*

**Figure 3:**
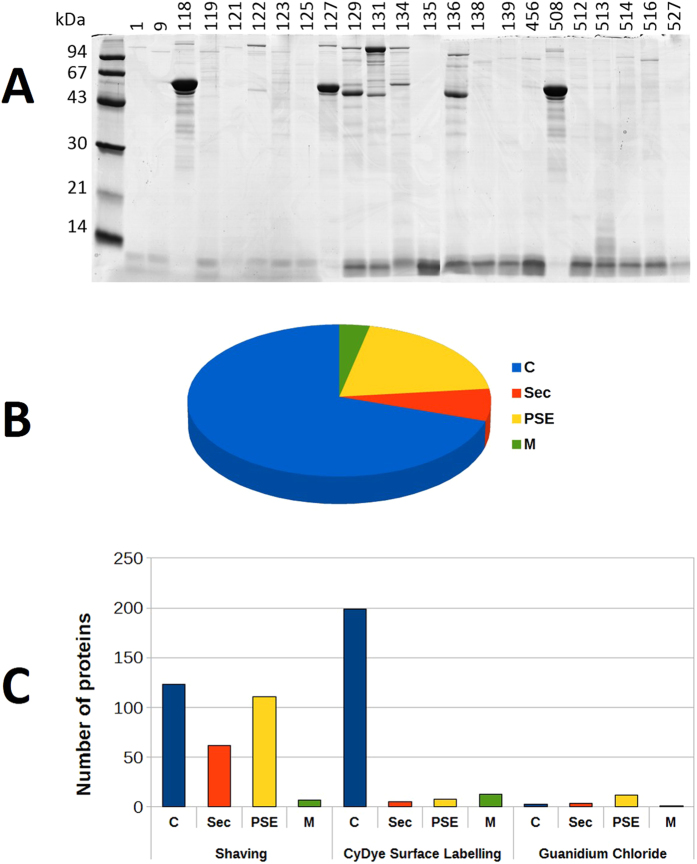
Diversity of *Propionibacterium freudenreichii* surface proteome and the distribution of identified surface proteins into *in silico* predicted localisations. (**A**) Guanidine hydrochloride-extracted proteins, analysed by SDS PAGE, evidenced variability among the *Pf* strains. (**B**,**C**). An inventory of the surface proteins of 12 *Pf* strains was further made by combining the three surface proteomic methods and produced a total of 509 proteomic identifications representing 174 different proteins. (**B**) Global distribution of *in silico* predicted localisations of the 174 different proteins identified. (**C**) Number and predicted localisations of the proteins identified using each analytical method: shaving, cyDye surface labelling and guanidine hydrochloride extraction (Le Maréchal *et al*.^19^). The localisations were predicted by SurfG + software, as described by Barinov *et al*.^20^ and the categories were as follows: (**C**) cytoplasmic protein, PSE: Protein surface exposed, M: membrane protein, SEC: secreted protein.

An initial global analysis of the proteins located at the surface of the cells, using guanidine hydrochloride extraction, revealed considerable diversity in terms of surface-layer-associated proteins, within the *P. freudenreichii* species (Fig. 3A). Thus, the surface proteomes of the 12 aforementioned strains were further inventoried using three different methods and compared to that of CIRM 129, which had previously been analysed under the same conditions^19^. All methods combined, the number of unique proteins identified for each strain ranged from 21 (CIRM 121) to 59 (CIRM 134). For the 12 strains, a total of 509 proteins were identified, representing 174 different proteins (many proteins were identified in several strains). Within these proteins, 23 could not be linked to a cluster because they corresponded to parts of bacterial genomes with poor quality sequences (inherent to draft genomes). About 70% of the 174 unique proteins identified were predicted as being cytoplasmic by SurfG + software^20^ (Fig. 3B). It should be noted that the number and identity of the proteins thus found was strongly dependent on the method used. As seen in Fig. 3C, proteins identified using the CyDye surface labelling method were mostly predicted as being cytoplasmic, so this method does not appear to be relevant when studying the surface proteome. However, with the shaving method, most of the proteins identified were predicted as being surface-exposed or secreted by SurfG+, which is consistent with their putative localisation. Among the 174 unique proteins, four were identified using the three methods in one or more strains. Three of them were surface proteins containing an SLH domain: SlpA (detected in CIRM118), SlpE (detected in CIRM129) and SlpB (detected in CIRM 118, 122 and 129). The fourth was LspA, a protein with a predicted molecular weight of 115 kDa and a glucosaminidase domain, which was detected in CIRM 456, 516 and 139. Finally, among the 174 proteins identified, one, represented by PFCIRM129_08670, was common to all 12 strains. The sequence of this protein contains a peptide signal and its predicted function is a secreted cell-wall peptidase. 77 proteins were identified in only one of the 12 strains, and could represent the specific proteome of each strain. All the other proteins identified were strain-dependent and could potentially be responsible for the strain-dependency of the anti-inflammatory properties of *Pf*. These proteomic data are summarised in the supplemental data online, Table S4.

#### Identification by association analysis of candidate genes potentially involved in the anti-inflammatory properties of *P. freudenreichii*

**Figure 4:**
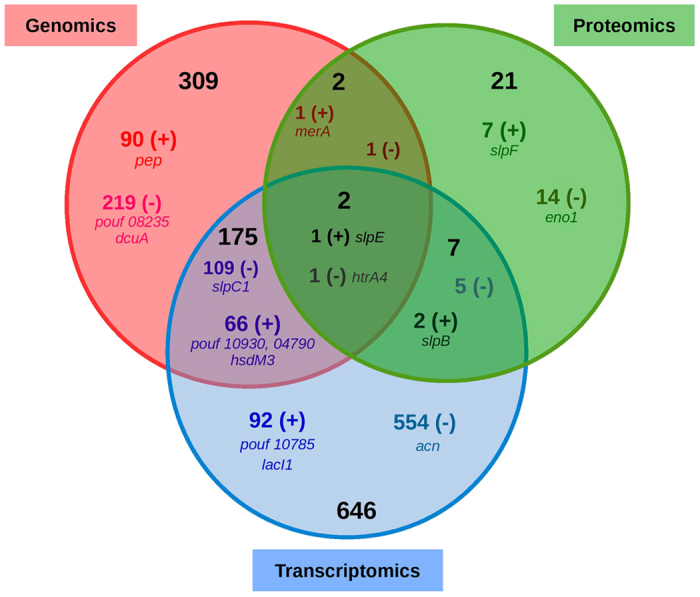
Venn diagram of *Propionibacterium freudenreichii* candidate genes identified as potentially being associated with anti-inflammatory properties, using omics methods (genomics, transcriptomics and surface proteomics). The numbers of candidates are those for which the regression model provides an adjusted p-value < 0.05 for the association. The regression model also provides a sign (+) or (−) corresponding to positive and negative associations with the induction of IL-10. (+) clusters are associated with IL-10 induction by anti-inflammatory strains whereas the reverse is true for (−) clusters. The names of the genes are specified for those mentioned in the text of the publication and in Table 1.

Reconciling the three types of data enabled the construction of a Venn diagram (Fig. 4). 488 clusters correlated with the IL-10 phenotype (adjusted p value < 0.05), 158 of them positively, based on their presence in or absence from the genomes of different strains. Likewise, 830 clusters were identified as being associated with the anti-inflammatory IL-10 phenotype, (p value < 0.05), 161 of them positively, based on the normalised expression levels of mRNA from those clusters. Finally, 32 clusters were found to be associated with the anti-inflammatory IL-10 phenotype, 11 of them positively, based on their presence in or absence from the surface proteomes of strains. Two clusters were identified as being associated with the anti-inflammatory phenotype by 3 omics typing: the clusters represented by *htrA4* (negative association) and *slpE* (positive association).

### Selection of candidate genes

The fifteen genes displaying the strongest association with IL-10 induction (five from genomics, five from transcriptomics, five from surface proteomics) were retained to validate their potential anti-inflammatory properties (see Table 1). The *groL2* gene, the fifth candidate under the proteomics approach, was associated with proteomic analysis (R^2^ = 0.20, adjusted p = 0.01) and with transcriptomic analysis (R^2^ = 0.05, not in the top five with this method) but was eliminated from the selection as it was ambiguous; being positively associated with transcriptomics and negatively with proteomics. As well as the previous 14 genes, two genes were added to the selection based on prior results: *slpB*, and *htrA4*. The *slpB* gene was positively associated in proteomics and transcriptomics, and the SlpB protein had been suspected of being anti-inflammatory in a previous work^19^. The *htrA4* gene was retained because it was associated with all three methods (adjusted p < 0.05), even though it was not in the top five with any of the methods. Within the 16 selected clusters, the representative genes *slpB* (PFCIRM129_00700), *slpE* (PFCIRM129_05460), *slpF* (PFCIRM129_01545) and *slpC1* (PFCIRM121_08040) encode cell wall proteins that are predicted as surface exposed proteins. All these proteins contain Pfam SLH domains (Surface Layer Homology domain) and were confirmed as being surface exposed by proteomic analysis. The *hsdM3* (PFCIRM129_00525), *merA* (PFCIRM129_03920) and *lacI1* (PFCIM129_07765) genes encode predicted cytoplasmic proteins, involved in DNA restriction and modification, coenzyme metabolism and the transcription of regulation, respectively. The *eno1* gene (PFCIRM121_10305) encodes a glycolytic enzyme, whereas *acn* encodes an enzyme of the TCA cycle responsible for the interconversion of citrate and isocitrate. The *dcuA* gene encodes a transport protein that is involved in C4-dicarboxylate transport, probably in the context of aspartate import. Finally, the *pep* gene encodes a peptidase. Four clusters were represented by genes encoding proteins of unknown function (Pouf): *pouf 10785, pouf 10930, pouf 04790* and *pouf 08235*. No homologies or protein domains for these genes were found in the databases.

### Inactivation of the selected candidate genes

**Figure 5:**
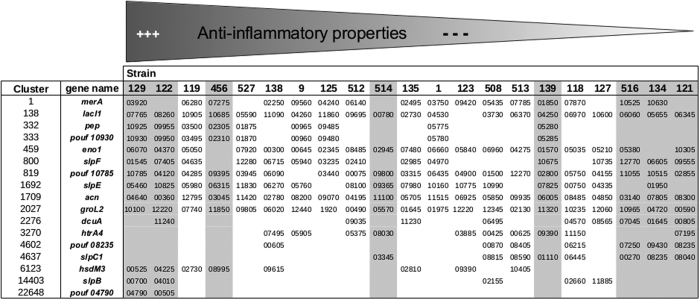
Identification of the *Propionibacterium freudenreichii* strains containing the genes coding for the candidate proteins identified by a multi-omics approach. The columns highlighted in grey correspond to the strains selected for the transcriptomic analysis. The numbers indicated in the Table refer to the locus tag of the genes; for example *merA* in strain CIRM 129 refers to the PFCIRM129_03920 gene.

In order to evaluate the role of the candidate genes in the anti-inflammatory properties of *Pf*, we pursued several strategies. When the regression coefficient was positive, inactivation of the candidate genes was attempted in *Pf* CIRM 129, the most anti-inflammatory strain. When the regression coefficient was negative, inactivation was attempted in *Pf* CIRM 121, the least anti-inflammatory strain. Despite numerous trials, we did not succeed in inactivating the following candidate genes: *merA, lacI1, pouf 10785, pouf 10930* and *pouf 04790* in *Pf* CIRM 129, and *acn, slpC1* and *dcuA* in *Pf* CIRM 121. For some of these genes, we cannot exclude that their inactivation was lethal, thus making it impossible to obtain a mutant. Furthermore, in *Pf* CIRM 121, our experiments led us to conclude that the process of homologous recombination, that should have been specific, was not so in some cases. As a consequence, we observed a non-specific and random recombination of the pUC vector in the chromosomic DNA of the strain. Finally, the *slpB* (PFCIRM129_00700), *slpE* (PFCIRM129_05460), *slpF* (PFCIRM129_01545), *hsdM3* (PFCIRM129_00525) and *pep* (PFCIRM129_10925) genes were successfully inactivated in *Pf* CIRM129, while the *htrA4* (PFCIRM121_07195), *eno1* (PFCIRM121_10305) and *pouf* 08235 (PFCIRM121_08235) genes were successfully inactivated in *Pf* CIRM 121 (Fig. 5).

### *In vitro* validation of mutants on human PBMCs

**Figure 6:**
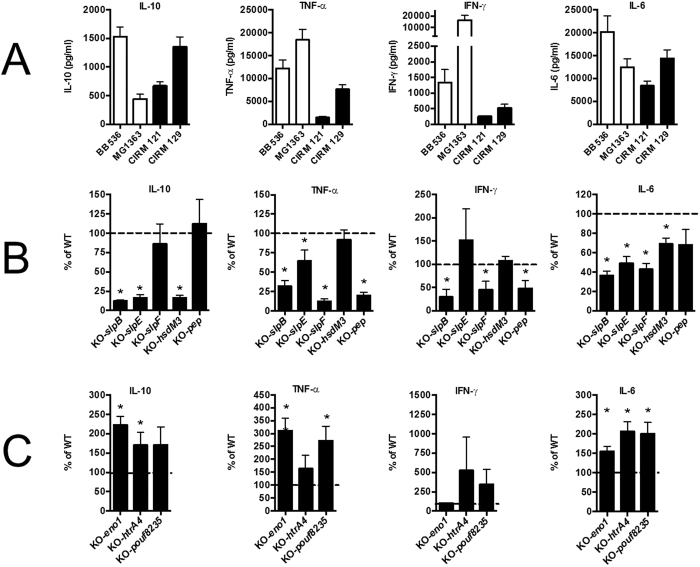
Immunological consequences of the inactivation of selected genes. Panel (A) Comparative IL-10, TNF-α, IFN-γ and IL-6 cytokine responses of human PBMCs stimulated by the two strains used as positive controls *Lactococcus lactis* MG1363 (pro-inflammatory) and *Bifidobacterium longum* BB536 (anti-inflammatory) or by two wild type *Propionibacterium freudenreichii* strains, CIRM 121 and CIRM 129. Panel (B) Cytokine induction by the mutant strains inactivated for the five genes positively associated with IL-10: *slpB, slpE, slpF, hsdM3* and *pep*, expressed as a percentage of the strong anti-inflammatory wild type CIRM 129 strain. Panel C. Cytokine induction by mutant strains inactivated for *eno1, htrA4, pouf8235*, negatively associated with IL-10, and expressed as a percentage of the weak anti-inflammatory wild type CIRM 121 strain. Data were expressed as means +/− SEM. Symbols: *P < 0.05.

The different mutant strains were tested for their immuno-modulatory properties on PBMCs and were compared to the corresponding wild type strains (Fig. 6). In *Pf* CIRM 129, the inactivation of *slpB, slpE* and *hsdM3* led to a marked reduction in the secretion by PBMCs of the anti-inflammatory cytokine IL-10, whereas the inactivation of *slpF* and *pouf 10925* produced no significant effect. This result demonstrated that *slpB, slpE* and *hsdM3* are involved in the anti-inflammatory properties of the CIRM 129 strain. Secretion of the dual (pro- and anti-inflammatory) IL-6 and pro-inflammatory TNF-α and IFN-γ, cytokines was also analysed. A decreased secretion of IL-6 was measured with *slpB, slpE, hsdM3* and *slpF* mutant strains compared to the WT strain. The inactivation of *slpB, slpF* and *pouf 10925* led to the reduced secretion of both TNF-α and IFN-γ, whereas the inactivation of *slpE* only decreased the secretion of TNF-α. The inactivation of *hsdM3* had no effect on the secretion of pro-inflammatory cytokines. Overall, these results demonstrated: i) the involvement of SlpB and SlpE in the secretion by PBMCs of both anti- and pro-inflammatory cytokines in the strain *Pf* CIRM 129, ii) the involvement of HsdM3 in the secretion of anti- inflammatory cytokines, and iii) the involvement of SlpF and Pep in the *Pf* CIRM 129-induced secretion of pro-inflammatory cytokines.

In *Pf* CIRM 121, mutations of *eno1, htrA4* and *pouf 08235* tended to increase, or significantly increased, the secretion of anti- and pro-inflammatory cytokines (IL-10, TNF-α and IL-6). Nevertheless, these mutations did not impact the secretion levels of IFN-γ, which are very low or non-existent with *Pf* CIRM 121. Collectively, these results showed the involvement of *eno1, htrA4* and *pouf 08235* in the weak secretion by PBMCs of *Pf* CIRM 121-induced pro- and anti-inflammatory cytokines.

## Discussion

Dietary components with immunomodulatory properties, including bacteria, have recently attracted considerable attention. In particular, probiotic bacteria with anti-inflammatory capabilities offer new perspectives in the context of a rising incidence of pathologies linked with dysbiosis^21^. The latter involves an imbalance of the gut microbiota, involving the expansion of pro-inflammatory bacteria at the expense of their anti-inflammatory counterparts^22^. Diet modulates the structure, diversity and metabolism of the gut microbiota^23^,^24^, suggesting that these diseases could be alleviated by dietary intervention. In this context, numerous studies have addressed the immunological status of bacteria ingested *via* food or as a probiotic supplement. The underlying motivation is that manipulating the gut microbiota *via* food-borne bacteria offers a promising approach to preventing and/or treating pathologies linked with dysbiosis. Dairy propionibacteria form a rich group of bacterial species, with diverse beneficial, technological and health properties, such as vitamin B12 synthesis, the biosynthesis of bifidogenic compound such as DHNA, or the synthesis of aroma compounds in cheese^1^. A specific feature of the *P. freudenreichii* species is the overall anti-inflammatory properties of its strains, although to varying degrees, which have been observed both *in vitro*^7^ and *in vivo* in humans and animal models^10^,^11^,^25^. During the present work, we confirmed the strain-dependent anti-inflammatory effect of *Pf* when grown on a dairy medium, and we evidenced a variable strain-dependent surface proteome. We postulate that the correlation between these two variabilities (proteome and immunomodulation) may lead to identification of the protein(s) involved. In order to decipher the molecular components associated with anti-inflammatory strains of the *Pf* species, we implemented a strategy that combined three levels of analysis: genomics, transcriptomics and surface proteomics. Such a strategy has never previously been described in the literature, many studies having focused on the role of specific surface components in these anti-inflammatory effects. As an example, several lactobacillus species are able to produce S-layers, a crystalline array constituted of self-assembling S-layer proteins subunits^26^. These proteins are often identified as immunomodulatory components. Indeed, purified SlpA of *L. acidophilus* NCFM was demonstrated as being responsible for the anti-inflammatory cytokine induction profile of the strain, because the protein induced higher levels of IL-10 in the presence of LPS^27^. In the same way, S-layer protein A of *L. helveticus* MIMLh5 was demonstrated to exert pro-inflammatory effects on immune cells^28^. Therefore, not all the S-layer proteins of lactobacilli are anti-inflammatory. For example, in *L. helveticus* NS8, the S-layer protein was not found to exert any anti-inflammatory properties after extraction^29^. Other studies identified different cell wall components as being mediators of the anti-inflammatory properties of bacterial strains, such as the peptidoglycan in *Lactobacillus salivarius* Ls33^30^, or teichoic acids in *Lactobacillus plantarum* NCIMB8826^31^.

A few *Pf* strains are covered by an abundant proteinaceous paracrystalline surface layer (S-layer), while others are not^32^. Moreover, the surface of *Pf* strains may also be covered with different proteins not organized as an S-layer. In a previous work, we identified five surface proteins as potentially being implicated in the strong anti-inflammatory properties of the *Pf* CIRM 129 strain^19^. These surface proteins, extracted by guanidium hydrochloride and identified by mass spectrometry analysis, were InlA, LspA, SlpE, SlpA and SlpB. In the present work, using mutant strains inactivated in the *slpB* and *slpE* genes, we confirmed unambiguously the key role of SlpB and SlpE in these anti-inflammatory properties. The InlA, LspA and SlpA proteins are probably not implicated, as they were not highlighted by our multi-approach analysis. SlpF, another surface protein containing an SLH domain, was pointed out by our strategy. However, its anti-inflammatory role was not confirmed by the gene-inactivation experiments in strain CIRM 129, so this is not implicated for this strain. We cannot however exclude that this protein might have an effect in another strain. As mentioned before for different lactic acid bacteria species, some surface components of *Pf* have been demonstrated to mediate the anti-inflammatory properties of the species. However, unsuspectingly, our strategy led to the identification of a cytoplasmic protein with an anti-inflammatory effect: HsdM3. This protein, involved in the DNA repair system, has never previously been identified as being anti-inflammatory, and further investigations are now necessary to clarify its role. Proteins predicted as having a cytoplasmic localisation and function have already been described as moonlighting, i.e. having a secondary surface localisation and effects on host immune responses (adhesion or immunomodulation)^33^.

Furthermore, and although SlpB and SlpE have been shown unambiguously to play a key role in the anti-inflammatory properties of *Pf*, they are not the exclusive drivers. Indeed, the *slpB* gene was present in the two most anti-inflammatory strains CIRM 129 and CIRM 122 and the corresponding protein was detected at the surface of these strains by proteomic analysis. However, *slpB* was missing from strains CIRM 119 and 456, which also exhibit strong anti-inflammatory properties (Fig. 5). Moreover, the SlpB protein was detected at the surface of the CIRM 118 strain, even though that strain is weakly anti-inflammatory (Fig. 5). The *slpE* gene was present in all the strains except for CIRM 125 and 513 and the two non-anti-inflammatory strains 516 and 121 (see Fig. 5). However, the SlpE protein was only detected in the surface proteome of CIRM 129 and not in the proteome of other strongly inflammatory strains. Because the transcriptomic data revealed a high level of *slpE* transcription in CIRM 129 (by comparison with other strains possessing the gene), strain-specific transcriptional regulation seems to explain the presence of the anti-inflammatory SlpE protein at the surface of *Pf*. Taken together, our results strongly suggest that the anti-inflammatory phenotype is due to a combination of different proteins that are mainly surface exposed. During this work, we identified the main actors explaining this phenotype. Thus, as has been demonstrated for lactobacilli and bifidobacteria^26^,^34^, the immunomodulatory response is the result of the detection by immunocompetent cells of a mosaic of surface proteins and other components (pro-and anti-inflammatory proteins). All these components contribute to the net balance of immunomodulatory properties of *Pf* strains. Finally, the presence of certain proteins identified as being negatively correlated must be taken into account as possibly playing a role in the global architecture of the cell wall, as was observed previously for the β-glucan polysaccharide produced by several strains of *Pf*^18^.

Overall, these results showed that different molecular mechanisms and/or components are at play in this context. There is not a single component in *Pf* that is responsible for the anti-inflammatory properties of all strains, but these anti-inflammatory properties result from a combination of not only surface components but also probably cytoplasmic actors. The relative contribution of each component differs from one strain to another and is still difficult to evaluate. In our study, we identified some drivers of anti-inflammatory properties of *Pf*, including surface proteins SlpB and SlpE. The validation of many of the other candidate genes highlighted by our multi-omics approach was however limited by the difficulties encountered in transforming and inactivating genes in *Pf*. The same difficulties prevented us from obtaining double mutant strains, a prerequisite for the biological validation of interactions between different components. Finally, our study demonstrated the natural biodiversity within the *Pf* species regarding its immunomodulatory properties. In terms of the development of probiotics, a promising option may be to screen all *Pf* strains and ensure careful association of strains with the different and complementary genes responsible for anti-inflammatory properties. This could lead to the development of new probiotic products for use in target populations with dysbiosis.

## Materials and Methods

### Bacteria and growth medium

The *Pf* strains and their genetically modified derivatives are listed in supplementary Table S1 online. The wild-type (WT) strains were provided by the CIRM-BIA Biological Resource Center (Centre International de Ressources Microbiennes-Bactéries d’Intérêt Alimentaire, INRA, Rennes, France). They were stored at −80 °C in YEL medium^35^. Before use, they were subcultured from frozen stock two times using a 2% inoculum either on YEL medium or on skim cow milk ultrafiltrat supplemented with 50 mM of sodium L-lactate (galaflow SL60, Société Arnaud, Paris, France) and 5 g/L of casein hydrolysate (Organotechnie, La Courneuve, France)^36^. The growth was performed at 30 °C without shaking. In some cases specified in the text, YEL was supplemented with chloramphenicol (10 μg/ml-1) or hygromycin B (750 μg/ml-1 in YEL agar or 250 μg/ml-1 in liquid YEL). The following bacterial strains of other species were used as positive controls for immune cell stimulation: *Lactococcus lactis* MG1363^37^ and *Bifidobacterium longum* BB536^38^. They were prepared as previously described^37^.

### *In vitro* immunomodulatory properties (PBMC)

Peripheral blood mononuclear cells (PBMCs) were isolated from peripheral blood of at least four healthy french donors as previously described^37^. Briefly, after a Ficoll gradient centrifugation (Pharmacia, Uppsala, Sweden), mononuclear cells were collected, washed in RPMI 1640 medium (Live technologies, Paisley, Scotland) and adjusted to 2 × 10^6^ cells/mL in RPMI 1640 supplemented with gentamicin (150 μg.mL) and 10% Fetal Calf Serum (Gibco-BRL). PBMCs (2 × 10^6^ cells/mL) were seeded in 48-well tissue culture plates (Corning, N.Y. US). Then, ten microliters of thawed bacterial suspensions of microorganisms to be tested and the positive control strains were added. The later were prepared as followed. Stationary-phase grown microbes were used, in order to be better aligned with the conditions encountered in fermented dairy foods. The microbes were washed twice in PBS, resuspended in PBS containing 20% glycerol using a portable photometer (Densimat, BioMérieux, Craponne, France) to adjust cell density to McFarland 3 and stored at −80 °C until further use^39^. These standardized bacterial preparations in terms of cell biomass, correspond to approximately 1–2 × 10^9^ CFU/mL, for *Pf* strains. This resulted in a bacteria-to-immune cell ratio of approximately 10:1. PBS containing 20% glycerol was used as a negative (non-stimulated) control. On the basis of preliminary time-course studies, 24 h stimulation corresponded to the best time point for cytokine responses of the PBMCs. After 24 h stimulation at 37 °C in an atmosphere of air with 5% CO_2_, culture supernatants were collected and we verified that most of the bacteria were alive by plating on YEL agar medium. The culture supernatants were clarified by centrifugation and stored at −20 °C until cytokine analysis. Neither medium acidification nor bacterial proliferation was observed. Cytokines were measured by ELISA using BD Pharmingen antibody pairs (BD Biosciences, San Jose, Ca, USA) for IL-10, IL-12 and IFN-γ and R&D systems Kit for IL-6 and TNF-α according to the manufacturer’s recommendations. Data were expressed as means +/− SEM, either in pg.mL^−1^ for cytokines, in relative proportion of the WT strain in the case of mutants, and in relative values for IL-10/IL-12 ratio.

### Statistical analysis

Results are expressed as the mean ± standard error of the mean (SEM). Statistical analyses were performed using GraphPad Prism 6 software (GraphPad Software Inc., La Jolla, CA, USA). Non- parametric Mann–Whitney tests were used to calculate significance levels for measurements. Values of P < 0.05 were considered statistically significant.

### Clustering of orthologous proteins based on whole genomes sequences

The whole genome sequencing, assembly and annotation of the 21 strains of *Pf* were conducted as previously described^6^. To establish the correspondence between the gene repertoires of the 21 *Pf* strains, we used single-linkage clustering whereby two predicted coding sequences were considered as belonging to the same gene cluster when blastp revealed a local alignment with at least 80% identity and accounting for at least 88% of the protein length (and E-value < 0.001). These thresholds were selected to maximize the number of clusters with exactly one representative per genome, i.e. single copy genes in the core genome. This approach led to the identification of 4971 clusters and the content of each genome was summarized as a presence/absence profile (across the 21 strains) of the 4971 genes (clusters). Based on contrasted IL-10 cytokine induction levels despite a high phylogenetic proximity, 8 out the 21 strains were selected for further transcriptomics analysis (CIRM 121, 122, 129, 134, 139, 456, 514, and 516) and 12 for proteomics analysis (adding CIRM 1, 118, 138 and 527). This choice minimized the confounding effect of phylogenetic inertia, i.e. the chance that a cluster is associated with high IL-10 induction levels simply because of shared evolutionary history of the 21 strains^40^.

### Phylogenetic analysis

A phylogenetic tree was reconstructed based on the concatenation of orthologous proteins of the core genome, *i*.e. proteins present in single copy in all strains. The phylogeny was reconstructed using PhyML (version 3) with the JTT + I + G model, selected by ProtTest. The inferred tree had high bootstrap values (>80) for all major clades with only 4 low values (<60), for shallow nodes. The topology was rooted at the midpoint of the longest distance between two strains in the 21-strain tree (midpoint rooting).

### Transcriptomic analysis

The 8 aforementioned strains were grown on skim cow milk ultrafiltrat supplemented with sodium L-lactate and casein hydrolysate for 72 h, at 30 °C. The RNA extraction was performed as previously described^41^ using Rneasy Mini Kit (Qiagen, Germany) and a subsequent DNase treatment (Dnase Rnase free, Ambion) according to the supplier. RNA concentrations were quantified using a Nanodrop. RNA quality (RIN) was evaluated using an Agilent 2100 bioanalyzer (Agilent Technologies, Santa Clara, CA). RNA labeling and hybridization were performed by the ‘Biopuces GeT plateforme’ (INSA Toulouse, France). All of the RNA samples had a RIN value greater than 8.3, indicating a good RNA integrity. The ratios 260/230 and 260/280 were greater than 1, 8.

A pangenomic 4 × 72 K Nimblegen microarray (Roche Diagnostics, France) was designed. For this purpose, we used the Oligowiz software^42^ and a reference pangenome composed of a representative sequence for each gene cluster to build a repertoire of possible isothermal probes of length between 45 nt and 65 nt. A relatively high target melting temperature 90 °C (+/−5 °C) was selected to account for the high G + C-content of *Pf* (67%). Then, we proceeded to the probe selection according to two criteria. First, we required at least 45 nt between the 5′-ends of two adjacent probes on the reference sequence (implying the overlap to be less than 20 nt). Second, we preferred probes corresponding to genomic regions without SNPs and discarded all probes whose sequences contained more than two mismatches between any of the 8 genomes and the reference pangenome. When SNPs existed, we included in the design a perfect match variant for each probe allele in the 8 genomes, and only perfect match variant was used to measure gene expression. For technical reasons, probes requiring more than 148 synthesis cycles were also filtered out. Finally, all probes were duplicated. The final design allowed to monitor the expression of 2,740 genes (clusters) based on probing 27,546 distinct genomic regions (only 594 genes found in at least 1 of the 8 strains were not covered). On average, the expression of a gene was monitored *via* 10 distinct probe sequences (corresponding to 20 measures of intensity since each probe was duplicated).

Retrotranscriptions, labelings, hydridizations and scans of the microarrays were then performed by the GeT-PlaGe platform (Genotoul, Toulouse, France) as follow. RNA were retrotranscribed into double stranded DNA using Superscript Double Strand cDNA Synthesis Kit (Invitrogen). The ratios 260/230 and 260/280 were greater than 1.8 and the concentrations greater than 150 ng/μl. According to the recommendations of the supplier, double stranded DNAs were labeled using One Color DNA Labeling (NimbleGen, Roche Diagnostics, France) and hybridizations were carried out using the NimbleGen Hybridization Kit and the Sample Tracking Control Kit (NimbleGen, Roche Diagnostics, France). Hybridized microarrays were scanned using a MS200 scanner (Roche Diagnostics, France) with a resolution of 5 μm.

The processing of the microarray data relied on custom Perl and R scripts. Measured intensities of the probes corresponding to the core genome (1345 genes) where quantile normalized towards a reference distribution obtained as a median over 16 hybridizations. The transformation specific to each array adjusted on the core genome was then also applied to the variable gene pool. For each hybridization, we computed an aggregated expression index for each gene in the corresponding strain as a median of the normalized intensities for the perfect match probes. After visual quality check based on the distribution of probe intensities, most strains were represented by two biological replicates (1 for CIRM 134, 3 for CIRM 514 and 516). Finally, the log2-transformed measures made on biological replicates were averaged for the subsequent correlation analysis with the immunological phenotype data. All microarray data are under submission with Gene Expression Omnibus (GEO; https://www.ncbi.nlm.nih.gov/geo/query/acc.cgi?token=yzenwcgivjslroj&acc=GSE87574).

### Proteomic analysis of bacterial surface

Surface proteins of several strains were detected using three complementary methods: trypsin-shaving, guanidine hydrochloride extraction and CyDye Cyanine surface labeling. All these methods were previously fully described in ref. 19.

### Association between anti-inflammatory phenotype of P. freudenreichii strains and the genomic, transcriptomic and proteomic data

The strategy used to associate genomic, transcriptomic and surface proteomic informations with anti-inflammatory phenotypes was to perform a regression study of phenotypes against omics features. Then candidates were selected based on the resulting adjusted p-values and R^2^s. The anti-inflammatory phenotype was based on IL-10 induction levels on PBMC responses, while predictors were omics information: presence/absence pattern for genomics and proteomics and expression levels for transcriptomics. The study was performed for each omics data type independently, before integrating the results from the 3 omics types to select the candidate genes for inactivation. For all omics types, we used linear mixed models to account for the multiplicity (x4) of PBMC donors. We regressed IL-10 induction levels against omics information (fixed effect), strain and donor (random effects); this is our full model. To assess the significance of the fixed effect, we also regressed IL-10 levels against strain and donor as random effects; this is our nested model with no omics effect but the same random effects as the full model. The p-value and fraction of variance explained (R^2^) by the omics effect were computed by comparing the full and nested models using either an ANOVA (p-value) or predicted IL-10 levels in both models (R^2^) (R functions: lmer from package lme4^43^ for the mixed models, anova and predict for the p-value and R-squared). The regression coefficient, which gives the direction (sign) and size of the omics effect, was recovered from the full model. Each cluster of orthologues for which omics information was available was tested individually and we corrected p-values using the fdr procedures to account for multiplicity of testing.

### Selection of candidate genes to be inactivated

Following the genomic, transcriptomic and proteomic correlation analysis, the clusters of orthologous were ordered according their R^2^ (fraction of IL-10 variance explained) for each omics type. The clusters containing only one CDS from one strain were filtered out. This filter led to the elimination of the strain-specific genes (in other words, genes without homology in any of the other sequenced strains) to give priority to genes shared by at least two strains. The clusters are divided into two groups according to the sign of genomic, transcriptomic, proteomic regression coefficient. For each method, a positive sign means that, for all the strains having a representative in this cluster, the presence of the gene/protein or level of mRNA is positively correlated to the level of IL-10 production. A negative coefficient reflects the absence of the gene, protein or mRNA representative of the cluster in the strongly anti-inflammatory strains. Candidate genes were first filtered out to have coherent signs, and thus effects, across omics types. Then, the five candidate genes deemed most associated with IL-10 production levels (*i.e* highest R^2^) were chosen, omics type by omics type, for further validation.

### Construction of the genetically modified strains

The insertional inactivation of genes was conducted as previously described^17^. Briefly, a suicide vector was constructed by inserting a chloramphenicol resistance gene in a pUC18 plasmid. In the resulting pUC:CmR plasmid, an internal fragment of about 500-bp of the coding sequence of the candidate gene of the strain of interest was cloned. The strains of *Pf* to be tested were transformed with the resulting vector and transformants were selected on YEL agar medium with chloramphenicol. The insertion of the vector in the chromosome was checked by PCR detection of the chloramphenicol gene that should be positive. The stability of the insertion was checked in YEL without chloramphenicol. The plasmids used in this work are listed on line in the supplementary Table S2 and the sequences of the probes in the supplementary Table S3

## Additional Information

**How to cite this article:** Deutsch, S.M. *et al*. Identification of proteins involved in the anti-inflammatory properties of *Propionibacterium freudenreichii* by means of a multi-strain study. *Sci. Rep.* **7**, 46409; doi: 10.1038/srep46409 (2017).

**Publisher's note:** Springer Nature remains neutral with regard to jurisdictional claims in published maps and institutional affiliations.

## Supplementary Material

Supplementary Tables

## Figures and Tables

**Table 1:** Candidate genes possibly responsible for the anti-inflammatory properties of *P. freudenreichii.*

Gene locus tag	Gene name	Localisation^a^	Ranking^b^	Correlation^c^			Function/product	Inactivation^d^
				G	T	P		
PFCIRM121_10305	*eno1*	C	P1			(−) 0.4	Main glycolytic pathways/EC number 4.2.1.11, glycolysis enzyme, 2-Phospho-D-glycerate < = > Phosphoenolpyruvate + H_2_O	Yes
PFCIRM129_03920	*merA*	C	P2	(+) 0.079		(+) 0.245	Metabolism of coenzymes and prothetic groups/Pyridine nucleotide-disulphide oxidoreductase	No
PFCIRM129_05460	*slpE*	PSE	P3	(+) 0.030	(+) 0.148	(+) 0.205	Cell wall/surface protein containig 3 SLH domains predicted by HMM Pfam	Yes
PFCIRM129_01545	*slpF*	PSE	P4			(+) 0.205	Cell wall/surface layer protein containing 3 SLH domains predicted by HMM Pfam	Yes
PFCIRM129_10100	*groL2*	C	P5	(+) 0.052		(−) 0.205	Protein Folding/protein chaperon	Not tested
PFCIRM129_10785	*pouf 10785*	PSE	T1		(+) 0.761		No function/No homology found in the databases	No
PFCIRM121_08040	*slpC1*	PSE	T2	(−) 0.231	(−) 0.745		Cell wall/surface protein containing 1 SLH domain predicted by HMM Pfam	No
PFCIRM129_00525	*hsdM3*	C	T3	(+) 0.175	(+) 0.744		DNA restriction and modification/Type I site- specific DNA methylase	Yes
PFCIRM129_07765	*lacl1*	C	T4		(+) 0.693		Transcription regulation/transcriptional regulator, arabinose operon epressor	No
PFCIRM121_08300	*acn*	C	T5		(−) 0.683		TCA cycle/Aconitase	No
PFCIRM129_10930	*pouf 10930*	C	G1	(+) 0.263	(+) 0.518		No function/No homology found in the databases	No
PFCIRM129_10925	*pep*	C	G2	(+) 0.263			Metabolism of amino acids and related molecules/peptidase	Yes
PFCIRM129_04790	*pouf 04790*	C	G3	(+) 0.261	(+) 0.603		No function/No homology found in the databases	No
PFCIRM121_08235	*pouf 08235*	PSE	G4	(−) 0.245			No function/Highly conserved among bacteria	Yes
PFCIRM121_00805	*dcuA*	M	G5	(−) 0.232			Transport or binding of amino-acids/C4-dicarboxylates transport activity	No
PFCIRM129_00700	*slpB*	PSE	S1	(+) 0.014	(+) 0.624	(+) 0.122	Cell wall/surface protein containing 3 SLH domains predicted by HMM Pfam	Yes
PFCIRM121_07195	*htrA4*	SEC	S2	(−) 0.104	(−) 0.130	(−) 0.136	Trypsine-like serine protease	Yes

The localisation corresponds to the category predicted in silico with SurfG + software, as described by Barinov *et al*.^20^. C cytoplasmic protein, PSE: Protein surface exposed, M: membrane protein, SEC: secreted protein. ^b^Gene [P/T/G]i corresponds to the I-th candidate gene according to proteomic, transcriptomic and genomic data analysis. S1 and S2 correspond to genes selected for external reasons. ^c^correlation: G, T and P correspond to the R squared values (when available) of a regression of IL-10 production against a gene presence/absence pattern (G), expression levels (T) and protein presence/absence pattern at the bacterial surface (P). Pouf: Protein of Unknown Function. ^d^Means ‘genes successfully inactivated’. For details of the association study, see Methods section.
