# Supplementary material for: Identification of proteins involved in the anti-inflammatory properties of Propionibacterium freudenreichii by means of a multi-strain study
Source: Sci Rep. 2017 Apr 13;7:46409. doi: 10.1038/srep46409 (PMC5390290; doi:10.1038/srep46409)
Supplement: Supplementary Tables [file srep46409-s1.pdf]

Supplementary Data

Identification of proteins involved in the anti-inflammatory properties of *Propionibacterium freudenreichii* by means of a multi-strain study

Stéphanie-Marie Deutsch[1*], Mahendra Mariadassou[2], Pierre Nicolas[2], Sandrine Parayre[1], Rozenn Le Guellec[1], Victoria Chuat[1], Vincent Peton[1], Caroline Le Maréchal[1], Julien Burati[2], Valentin Loux[2], Valérie Briard-Bion[1], Julien Jardin[1], Coline Plé[3], Benoît Foligné[3], Gwénaël Jan[1$] & Hélène Falentin[1$]

[*]Corresponding author

contact information of corresponding author: stephanie-marie.deutsch@inra.fr

[$]share credit as senior co-authors

[1]STLO, UMR1253, INRA, Agrocampus Ouest, 35000, Rennes, France

[2]MaIAGE, UR1404, INRA, 78352 Jouy-en-Josas, France

[3]Univ. Lille, CNRS, Inserm, CHU Lille, Institut Pasteur de Lille, U1019 – UMR 8204 - CIIL - Center for Infection and Immunity of Lille, 59000 Lille, France

Supplementary Data

Supplementary Table S1: Strains of *Propionibacterium freudenreichii* and their derivatives

The sequences are deposited in the EMBL database and are publicy available. NS: not sequenced. CIRM-BIA: Centre International de Ressources Microbiennes – Bactéries d'Intérêt Alimentaire, INRA, UMR 1253, Science et Technologie du Lait et de l'Œuf, Rennes, France – abbreviated in the texte as "CIRM". ITG: Actalia collection (Rennes, France). LS: Laboratoire Standa Collection (Laboratoires Standa, Caen, France)

Strain or plasmid	Other Name	Relevant genotype and phenotype	Reference	Bioproject number
P. freudenreichii				
CIRM-BIA 1		Wild type, type strain	Falentin et al. 2010	FN806773
CIRM-BIA 9		Wild type	Loux et al. 2015	PRJEB6427
CIRM-BIA 118		Wild type	Loux et al. 2015	PRJEB6428
CIRM-BIA 119		Wild type	Loux et al. 2015	PRJEB6430
CIRM-BIA 121		Wild type	Loux et al. 2015	PRJEB6431
CIRM-BIA 122		Wild type	Loux et al. 2015	PRJEB6432
CIRM-BIA 123		Wild type	Loux et al. 2015	PRJEB6438
CIRM-BIA 125	ITG P14	Wild type	Loux et al. 2015	PRJEB6434
CIRM-BIA 127	ITG P18	Wild type	Loux et al. 2015	PRJEB6435
CIRM-BIA129	ITG P20	Wild type	Le Maréchal et al	PRJEB4826
CIRM-BIA 131	LS 408	Wild Type	This work	Not available
CIRM-BIA134		Wild type	Loux et al. 2015	PRJEB6441
CIRM-BIA135		Wild type	Loux et al. 2015	PRJEB6442
CIRM-BIA 136	TL 1348	Wild Type	This work	Not available
CIRM-BIA138	ITG P9	Wild type	Loux et al. 2015	PRJEB6433
CIRM-BIA139	ITG P23	Wild type	Loux et al. 2015	PRJEB6436
CIRM-BIA 456		Wild type	Loux et al. 2015	PRJEB6445
CIRM-BIA 508		Wild type	Loux et al. 2015	PRJEB6429

CIRM-BIA 512	Wild type	Loux et al. 2015	PRJEB6439
CIRM-BIA 513	Wild type	Loux et al. 2015	PRJEB6440
CIRM-BIA 514	Wild type	Loux et al. 2015	PRJEB6443
CIRM-BIA 516	Wild type	Loux et al. 2015	PRJEB6444
CIRM-BIA 527	Wild type	Loux et al. 2015	PRJEB6437
CIRM-BIA129-KO-s*lpB*	Inactivation of the gene *slpB* in strain CIRM-BIA 129	This work	
CIRM-BIA129-KO-s*lpE*	Inactivation of the gene *slpE* in strain CIRM-BIA 129	This work	
CIRM-BIA129-KO-*slpF*	Inactivation of the gene *slpF* in strain CIRM-BIA 129	This work	
CIRM-BIA129-KO-*hsdM3*	Inactivation of the gene *hsdM3* in strain CIRM-BIA 129	This work	
CIRM-BIA129-KO-*pep*	Inactivation of the gene *pouf10925* in strain CIRM-BIA 129	This work	
CIRM-BIA121-KO-*eno1*	Inactivation of the gene *eno1* in strain CIRM-BIA 121	This work	
CIRM-BIA121-KO-*htrA4*	Inactivation of the gene *htrA4* in strain CIRM-BIA 121	This work	
CIRM-BIA121-KO-*pouf8235*	Inactivation of the gene *pouf8235* in strain CIRM-BIA 121	This work	

Supplementary Table S2: Plasmids used in the work

Plasmids	Genotype	Reference
pPK705	*E. coli-P. freudenreichii* shuttle vector, carrying ampicillin resistance gene	Kiatpapan et al. 2000
pUC:CmR	pUC18 carrying chloramphenicol resistance gene	Deutsch et al. 2010
PUC:CmR:*slpB*	pUC18 carrying chloramphenicol resistance gene and a truncated fragment of *slpB* of strain CIRM-BIA 129	This work
PUC:CmR:*slpE*	pUC18 carrying chloramphenicol resistance gene and a truncated fragment of *slpE* of strain CIRM-BIA 129	This work
PUC:CmR:*slpF*	pUC18 carrying chloramphenicol resistance gene and a truncated fragment of *slpF* of of strain CIRM-BIA 129	This work
PUC:CmR:*hsdM3*	pUC18 carrying chloramphenicol resistance gene and a truncated fragment of *hsdM3* of strain CIRM-BIA 129	This work
PUC:CmR:*pep*	pUC18 carrying chloramphenicol resistance gene and a truncated fragment of *slpE* of strain CIRM-BIA 129	This work
PUC:CmR:*eno1*	pUC18 carrying chloramphenicol resistance gene and a truncated fragment of *eno1* of strain CIRM-BIA 121	This work
PUC:CmR:*htrA4*	pUC18 carrying chloramphenicol resistance gene and a truncated fragment of *htrA4* of strain CIRM-BIA 121	This work
PUC:CmR:*pouf8235*	pUC18 carrying chloramphenicol resistance gene and a truncated fragment of *pouf8235* of strain CIRM-BIA 121	This work

Supplementary Table S3: Primers used in the study

Name	Sequence (5' to 3')	Size of amplicon (bp)	Application
PFCIRM129_00700_slpB_HindIII_F	ACACAAAGCTTAACTACTCGCCCACCAACAC	574	Amplification of the internal fragment of the gene to be inactivated
PFCIRM129_00700_slpB_HindIII_R	ACACAAAGCTTCACTCAGGCGGTAGAGGAAC		
PFCIRM129_05460_slpE_HindIII_F	ACACAAAGCTTCTCTGGAACCAGCGAGTAGC	570	
PFCIRM129_05460_slpE_HindIII_R	ACACAAAGCTTCTTCGCCCAGCAAATATCAT		
PFCIRM129_00525_hsdM3_HindIII_F	ACACAAAGCTTGTCCGACTACTCGCAACACA	599	
PFCIRM129_00525_hsdM3_HindIII_R	ACACAAAGCTTGGAACAACAGCTCCGTCTTC		
PFCIRM129_10925_pep_HindIII_F	ACACAAAGCTTATTCCGACGATGACTTCCAC	553	
PFCIRM129_10925_pep_HindIII_R	ACACAAAGCTTATAGGTGTGAACGGGCTGAC		
PFCIRM129_01545_slpF_HindIII_F	ACACAAAGCTTACGGCTATGACACCGAGTTC	544	
PFCIRM129_01545_slpF_HindIII_R	ACACAAAGCTTGTGATCTTCTGCCCATCGTT		
PFCIRM121_10305_eno1_HindIII_F	ACACAAAGCTTGAGGTTGAGGTCGTTCTGGA	586	
PFCIRM121_10305_eno1_HindIII_R	ACACAAAGCTTCACGGTTGTTGTCGAGGTTG		
PFCIRM121_08235_pouf_HindIII_F	ACACAAAGCTTGACACCTACGCCGAGTTGAT	531	
PFCIRM121_08235_pouf_HindIII_R	ACACAAAGCTTGTGCTCGACCTTCTTCTTGC		
PFCIRM121_07195_htrA4_HindIII_F	ACACAAAGCTTTCGTGCTGACGAACTACCAC	513	
PFCIRM121_07195_htrA4_HindIII_R	ACACAAAGCTTCCGAAGGTTCATTCCTTGAG		
Cm_F-NsiI	ACACAATGCATGGGTCATCAATTGGCCTC	1513	PCR detection of the chloramphenicol gene
Cm_R-NsiI	ACACAATGCATCGTCACACCCGAACATGTCG	1513	

The underlined sequences indicate the position of the restriction sites

Deutsch et al.,
Identification of proteins involved in the anti-inflammatory properties
of Propionibacterium freudenreichii by means of a multi-strain study

Supplementary Table S4: surface proteome of 12 strains of *Propionibacterium freudenreichii* identified by nano -LC-MS/MS*.*
Proteins were identified after *in situ* fluorescence labelling using CyDye DIGE Fluor Cy5 minimal dye (CyDye)
or after enzymatic shaving with trypsin (Shaving) or after Guanidine Hydrochloride extraction (ClGua).
Proteins molecular weights were automatically predicted from the corresponding genes on the Agmial annotation platform.
Coverage of a protein is calculated as the percentage of the amino acid sequence included in the identified peptides.
e-value is the number of times a given peptide score will be achieved by incorrect matches from a database search.
Protein identifications were automatically validated when they showed at least two unique peptides with an e-value below 0.05 corresponding to log(e-value) < -1.30

Locus Tag	Cluster	Method	Description	Function	Gene name	SurfG+ Predicted Localization	Molecular Weight (Da)	log(E value)	Coverage (%)	Number Of Unique Peptides
PFCIRM129_03920	**1**	**CyDye**	Pyridine nucleotide-disulphide oxidoreductase	Metabolism of coenzymes and prosthetic groups	**merA**	CYTO	48,100	-17,222	17	5
PFCIRM456_07275	**1**	**CyDye**	Pyridine nucleotide-disulphide oxidoreductase	Metabolism of coenzymes and prosthetic groups	**merA**	CYTO	48,200	-49,444	38	12
PFCIRM129_11210	**31**	**CyDye**	ABC-type transport involved in Fe-S cluster assembly, ATPase component, SufC	Transport/binding proteins and lipoproteins	**sufC**	CYTO	26,800	-25,249	30	6
PFCIRM134_03605	**31**	**CyDye**	ABC-type transport involved in Fe-S cluster assembly, ATPase component, SufC	Transport/binding proteins and lipoproteins	**sufC**	CYTO	26,800	-36,356	48	8
PFCIRM139_12255	**31**	**CyDye**	ABC-type transport involved in Fe-S cluster assembly, ATPase component, SufC	Transport/binding proteins and lipoproteins	**sufC**	CYTO	26,800	-34,713	41	7
PFCIRM516_00960	**33**	**Shaving**	FeS assembly protein SufD	Transport/binding proteins and lipoproteins	**sufD**	CYTO	43,800	-7,985	6	2
PFCIRM122_11925	**34**	**CyDye**	FeS assembly protein SufB		**sufB**	CYTO	53,800	-78,041	51	20
PFCIRM129_11225	**34**	**CyDye**	FeS assembly protein SufB		**sufB**	CYTO	53,800	-35,005	31	8
PFCIRM134_03620	**34**	**CyDye**	FeS assembly protein SufB		**sufB**	CYTO	53,800	-89,249	59	22
PFCIRM1_03980	**34**	**CyDye**	FeS assembly protein SufB		**sufB**	CYTO	53,800	-44,205	31	11
PFCIRM118_03895	**34**	**CyDye**	FeS assembly protein SufB		**sufB**	CYTO	53,800	-127,340	66	23
PFCIRM138_01515	**34**	**CyDye**	FeS assembly protein SufB		**sufB**	CYTO	53,800	-51,576	31	11
PFCIRM122_11855	**47**	**CyDye**	triosephosphate isomerase 1	Main glycolytic pathways	**tpi1**	CYTO	27,500	-36,419	47	9
PFCIRM139_12175	**47**	**CyDye**	triosephosphate isomerase 1	Main glycolytic pathways	**tpi1**	CYTO	NA	-41,988	NA	10
PFCIRM1_04045	**47**	**CyDye**	triosephosphate isomerase 1	Main glycolytic pathways	**tpi1**	CYTO	27,500	-23,002	26	4
PFCIRM1_04050	**48**	**CyDye**	Phosphoglycerate kinase	Main glycolytic pathways	**pgk**	CYTO	42,300	-15,754	8	2
PFCIRM118_03965	**48**	**CyDye**	Phosphoglycerate kinase	Main glycolytic pathways	**pgk**	CYTO	42,300	-80,545	48	14
PFCIRM138_01585	**48**	**CyDye**	Phosphoglycerate kinase	Main glycolytic pathways	**pgk**	CYTO	42,300	-33,513	21	6
PFCIRM122_11845	**49**	**CyDye**	Glyceraldehyde-3-phosphate dehydrogenase Erythrose 4 phosphate dehydrogenase	Main glycolytic pathways	**gap**	CYTO	37,700	-10,608	11	3
PFCIRM138_01590	**49**	**CyDye**	Glyceraldehyde-3-phosphate dehydrogenase Erythrose 4 phosphate dehydrogenase	Main glycolytic pathways	**gap**	CYTO	37,700	-15,688	16	4
PFCIRM134_03795	**69**	**Shaving**	30S ribosomal protein S1	Ribosomal proteins	**rpsA**	CYTO	53,500	-6,574	5	2
PFCIRM456_08210	**70**	**CyDye**	Aldehyde dehydrogenase (Succinate-semialdehyde dehydrogenase)	Metabolism of carbohydrates And related molecules	**gab**	CYTO	52,200	-14,281	11	4
PFCIRM118_04075	**70**	**CyDye**	Aldehyde dehydrogenase (Succinate-semialdehyde dehydrogenase)	Metabolism of carbohydrates And related molecules	**gab**	CYTO	52,200	-63,918	42	12
PFCIRM129_11455	**78**	**CyDye**	Hypothetical protein	Pouf similar to proteins from other organisms		CYTO	36,400	-60,186	34	11
PFCIRM134_03845	**78**	**CyDye**	Hypothetical protein	Pouf similar to proteins from other organisms		CYTO	36,400	-44,611	32	11
PFCIRM516_01190	**78**	**CyDye**	Hypothetical protein	Pouf similar to proteins from other organisms		CYTO	36,400	-39,465	27	8
PFCIRM118_04120	**78**	**Shaving**	Hypothetical protein	Pouf similar to proteins from other organisms		CYTO	36,400	-26,584	27	5
PFCIRM129_11455	**78**	**Shaving**	Hypothetical protein	Pouf similar to proteins from other organisms		CYTO	36,400	-11,347	8	2
PFCIRM134_03845	**78**	**Shaving**	Hypothetical protein	Pouf similar to proteins from other organisms		CYTO	36,400	-11,965	8	2
PFCIRM1_04205	**78**	**Shaving**	Hypothetical protein	Pouf similar to proteins from other organisms		CYTO	36,400	-12,537	13	3
PFCIRM134_03910	**91**	**Shaving**	Cell division protein FtsI (penicillin-binding protein 2)	Cell wall	**ftsI**	PSE	NA	-10,600	NA	2
PFCIRM514_04610	**91**	**Shaving**	Cell division protein FtsI (penicillin-binding protein 2)	Cell wall	**ftsI**	PSE	71,800	-5,917	4	2

Locus Tag	Cluster	Method	Description	Function	Gene name	SurfG+ Predicted Localization	Molecular Weight (Da)	log(E value)	Coverage (%)	Number Of Unique Peptides
PFCIRM121_01110	**111**	**Shaving**	Peptidase M23B family / metalloendopeptidase	Cell envelope and cellular processes		SECRETED	38,900	-28,569	27	6
PFCIRM122_11535	**111**	**Shaving**	Peptidase M23B family / metalloendopeptidase	Cell envelope and cellular processes		SECRETED	38,900	-26,024	27	6
PFCIRM134_04010	**111**	**Shaving**	Peptidase M23B family / metalloendopeptidase	Cell envelope and cellular processes		SECRETED	38,900	-9,303	10	2
PFCIRM139_11840	**111**	**Shaving**	Peptidase M23B family / metalloendopeptidase	Cell envelope and cellular processes		SECRETED	38,900	-24,734	20	4
PFCIRM456_08000	**111**	**Shaving**	Peptidase M23B family / metalloendopeptidase	Cell envelope and cellular processes		SECRETED	38,900	-44,876	34	9
PFCIRM514_04510	**111**	**Shaving**	Peptidase M23B family / metalloendopeptidase	Cell envelope and cellular processes		SECRETED	38,900	-16,534	22	5
PFCIRM516_01355	**111**	**Shaving**	Peptidase M23B family / metalloendopeptidase	Cell envelope and cellular processes		SECRETED	38,900	-10,381	18	3
PFCIRM527_00510	**111**	**Shaving**	Peptidase M23B family / metalloendopeptidase	Cell envelope and cellular processes		SECRETED	38,900	-33,801	27	7
PFCIRM1_04370	**111**	**Shaving**	Peptidase M23B family / metalloendopeptidase	Cell envelope and cellular processes		SECRETED	38,900	-25,288	28	6
PFCIRM138_01925	**114**	**CyDye**	Hypothetical protein	Pouf similar to proteins from other organisms		CYTO	35,800	-18,925	19	5
PFCIRM1_04560	**142**	**CyDye**	Cysteine synthase 1	Metabolism of amino acids and related molecules	**cys1**	CYTO	31,600	-16,295	20	5
PFCIRM118_06945	**142**	**CyDye**	Cysteine synthase 1	Metabolism of amino acids and related molecules	**cys1**	CYTO	31,600	-25,408	29	7
PFCIRM138_11065	**142**	**CyDye**	Cysteine synthase 1	Metabolism of amino acids and related molecules	**cys1**	CYTO	31,600	-28,502	29	7
PFCIRM527_05615	**142**	**CyDye**	Cysteine synthase 1	Metabolism of amino acids and related molecules	**cys1**	CYTO	31,600	-24,512	26	6
PFCIRM121_06290	**146**	**Shaving**	Hypothetical protein	Pouf, without similarity to other proteins		PSE	58,300	-8,429	7	3
PFCIRM122_08215	**146**	**Shaving**	Hypothetical protein	Pouf, without similarity to other proteins		PSE	58,600	-11,410	9	3
PFCIRM139_04205	**146**	**Shaving**	Hypothetical protein	Pouf, without similarity to other proteins		PSE	58,600	-9,710	8	3
PFCIRM527_05635	**146**	**Shaving**	Hypothetical protein	Pouf, without similarity to other proteins		PSE	58,600	-6,553	5	2
PFCIRM516_06130	**150**	**CyDye**	Inosine-5 -monophosphate dehydrogenase	Metabolism of nucleotides and nucleic acids	**guaB1**	CYTO	53,800	-42,000	24	8
PFCIRM1_04600	**150**	**CyDye**	Inosine-5 -monophosphate dehydrogenase	Metabolism of nucleotides and nucleic acids	**guaB1**	CYTO	53,800	-29,271	15	5
PFCIRM122_00690	**193**	**CyDye**	D-alanine--D-alanine ligase (D-alanylalanine synthetase)	Cell wall	**ddlA**	CYTO	NA	-6,018	NA	2
PFCIRM456_03370	**193**	**CyDye**	D-alanine--D-alanine ligase (D-alanylalanine synthetase)	Cell wall	**ddlA**	CYTO	40,400	-9,173	10	3
PFCIRM516_04805	**193**	**CyDye**	D-alanine--D-alanine ligase (D-alanylalanine synthetase)	Cell wall	**ddlA**	CYTO	40,400	-16,534	17	5
PFCIRM129_04355	**200**	**CyDye**	Sua5_yciO_yrdC	Translation initiation		CYTO	22,600	-15,358	16	3
PFCIRM456_02925	**231**	**Shaving**	Hypothetical protein	Pouf similar to proteins from other organisms		PSE	25,800	-7,029	12	2
PFCIRM139_08140	**241**	**Shaving**	Acetolactate synthase subunit small	Metabolism of amino acids and related molecules	**ilvN**	CYTO	54,100	-7,347	5	2
PFCIRM139_01090	**250**	**CyDye**	surface layer protein A (S-layer protein A)	Cell wall	**slpA**	PSE	58,300	-74,925	59	18
PFCIRM118_06465	**250**	**CyDye**	surface layer protein A (S-layer protein A)	Cell wall	**slpA**	PSE	58,500	-102,331	53	20
PFCIRM118_06465	**250**	**ClGua**	surface layer protein A (S-layer protein A)	Cell wall	**slpA**	PSE	58,500	-72,019	36	14
PFCIRM129_09350	**250**	**ClGua**	surface layer protein A (S-layer protein A)	Cell wall	**slpA**	PSE	58,300	-32,692	20	6
PFCIRM134_08215	**250**	**ClGua**	surface layer protein A (S-layer protein A)	Cell wall	**slpA**	PSE	58,300	-93,770	43	17
PFCIRM456_11555	**250**	**ClGua**	surface layer protein A (S-layer protein A)	Cell wall	**slpA**	PSE	58,400	-37,411	18	8
PFCIRM118_06465	**250**	**Shaving**	surface layer protein A (S-layer protein A)	Cell wall	**slpA**	PSE	58,500	-399,131	84	80
PFCIRM129_09350	**250**	**Shaving**	surface layer protein A (S-layer protein A)	Cell wall	**slpA**	PSE	58,300	-24,241	13	5
PFCIRM134_08215	**250**	**Shaving**	surface layer protein A (S-layer protein A)	Cell wall	**slpA**	PSE	58,300	-61,794	21	11
PFCIRM138_05200	**250**	**Shaving**	surface layer protein A (S-layer protein A)	Cell wall	**slpA**	PSE	NA	-42,300	NA	9
PFCIRM456_11555	**250**	**Shaving**	surface layer protein A (S-layer protein A)	Cell wall	**slpA**	PSE	58,400	-15,850	13	4

Locus Tag	Cluster	Method	Description	Function	Gene name	SurfG+ Predicted Localization	Molecular Weight (Da)	log(E value)	Coverage (%)	Number Of Unique Peptides
PFCIRM122_08190	**252**	**CyDye**	60 kDa chaperonin 1 (Protein Cpn60 1) (groEL protein 1) (Heat shock protein 60 1)	Protein folding	**groL1**	CYTO	56,100	-106,379	50	25
PFCIRM134_05590	**252**	**CyDye**	60 kDa chaperonin 1 (Protein Cpn60 1) (groEL protein 1) (Heat shock protein 60 1)	Protein folding	**groL1**	CYTO	NA	-136,580	NA	31
PFCIRM139_04180	**252**	**CyDye**	60 kDa chaperonin 1 (Protein Cpn60 1) (groEL protein 1) (Heat shock protein 60 1)	Protein folding	**groL1**	CYTO	NA	-10,336	NA	3
PFCIRM1_05320	**252**	**CyDye**	60 kDa chaperonin 1 (Protein Cpn60 1) (groEL protein 1) (Heat shock protein 60 1)	Protein folding	**groL1**	CYTO	56,000	-46,939	23	8
PFCIRM138_11020	**252**	**CyDye**	60 kDa chaperonin 1 (Protein Cpn60 1) (groEL protein 1) (Heat shock protein 60 1)	Protein folding	**groL1**	CYTO	56,000	-91,900	39	16
PFCIRM527_05660	**252**	**CyDye**	60 kDa chaperonin 1 (Protein Cpn60 1) (groEL protein 1) (Heat shock protein 60 1)	Protein folding	**groL1**	CYTO	56,000	-109,250	52	23
PFCIRM118_06900	**252**	**Shaving**	60 kDa chaperonin 1 (Protein Cpn60 1) (groEL protein 1) (Heat shock protein 60 1)	Protein folding	**groL1**	CYTO	56,000	-49,146	35	9
PFCIRM129_07835	**252**	**Shaving**	60 kDa chaperonin 1 (Protein Cpn60 1) (groEL protein 1) (Heat shock protein 60 1)	Protein folding	**groL1**	CYTO	56,000	-10,320	12	3
PFCIRM134_05590	**252**	**Shaving**	60 kDa chaperonin 1 (Protein Cpn60 1) (groEL protein 1) (Heat shock protein 60 1)	Protein folding	**groL1**	CYTO	56,100	-42,539	34	9
PFCIRM139_04180	**252**	**Shaving**	60 kDa chaperonin 1 (Protein Cpn60 1) (groEL protein 1) (Heat shock protein 60 1)	Protein folding	**groL1**	CYTO	56,000	-21,067	20	5
PFCIRM516_06135	**252**	**Shaving**	60 kDa chaperonin 1 (Protein Cpn60 1) (groEL protein 1) (Heat shock protein 60 1)	Protein folding	**groL1**	CYTO	56,100	-29,026	28	7
PFCIRM1_05320	**252**	**Shaving**	60 kDa chaperonin 1 (Protein Cpn60 1) (groEL protein 1) (Heat shock protein 60 1)	Protein folding	**groL1**	CYTO	56,000	-23,065	21	6
PFCIRM139_04175	**253**	**CyDye**	10 kDa chaperonin 1 (Protein Cpn10 1 (groES protein 1) (Heat shock 10 1)	Protein folding	**groS1**	CYTO	NA	-6,211	NA	2
PFCIRM139_04175	**253**	**Shaving**	10 kDa chaperonin 1 (Protein Cpn10 1) (groES protein 1) (Heat shock 10 1)	Protein folding	**groS1**	CYTO	10,500	-9,143	34	2
PFCIRM1_05325	**253**	**Shaving**	10 kDa chaperonin 1 (Protein Cpn10 1) (groES protein 1) (Heat shock 10 1)	Protein folding	**groS1**	CYTO	10,500	-9,153	42	2
PFCIRM118_06880	**256**	**CyDye**	ATP-binding protein opuCA of Glycine betaine/carnitine/choline ABC transporter	Transport/binding of amino-acids	**opuCA**	CYTO	48,000	-14,901	14	4
PFCIRM134_09550	**264**	**CyDye**	50S ribosomal protein L7/L12	Ribosomal proteins	**rplL**	CYTO	13,600	-17,173	35	5
PFCIRM456_10160	**264**	**CyDye**	50S ribosomal protein L7/L12	Ribosomal proteins	**rplL**	CYTO	13,600	-33,686	72	6
PFCIRM514_01645	**264**	**CyDye**	50S ribosomal protein L7/L12	Ribosomal proteins	**rplL**	CYTO	13,600	-34,306	77	9
PFCIRM118_01845	**264**	**Shaving**	50S ribosomal protein L7/L12	Ribosomal proteins	**rplL**	CYTO	15,700	-10,037	19	2
PFCIRM118_01870	**264**	**Shaving**	50S ribosomal protein L7/L12	Ribosomal proteins	**rplL**	CYTO	13,600	-6,054	18	2
PFCIRM134_09550	**264**	**Shaving**	50S ribosomal protein L7/L12	Ribosomal proteins	**rplL**	CYTO	13,600	-16,327	58	4
PFCIRM139_03695	**264**	**Shaving**	50S ribosomal protein L7/L12	Ribosomal proteins	**rplL**	CYTO	13,600	-9,265	37	3
PFCIRM139_03720	**264**	**Shaving**	50S ribosomal protein L7/L12	Ribosomal proteins	**rplL**	CYTO	17,400	-11,936	24	3
PFCIRM1_05390	**264**	**Shaving**	50S ribosomal protein L7/L12	Ribosomal proteins	**rplL**	CYTO	13,600	-7,307	18	2
PFCIRM122_06715	**269**	**CyDye**	Elongation factor G (EF-G)	Translation elongation	**fusA**	CYTO	76,500	-37,847	22	10
PFCIRM129_08280	**269**	**CyDye**	Elongation factor G (EF-G)	Translation elongation	**fusA**	CYTO	76,500	-37,976	18	9
PFCIRM134_09520	**269**	**CyDye**	Elongation factor G (EF-G)	Translation elongation	**fusA**	CYTO	76,500	-43,604	21	11

Locus Tag	Cluster	Method	Description	Function	Gene name	SurfG+ Predicted Localization	Molecular Weight (kDa)	log(E value)	Coverage (%)	Number Of Unique Peptides
PFCIRM122_06710	**270**	**CyDye**	Elongation factor Tu	Translation elongation	**tuf**	CYTO	43,600	-81,218	44	14
PFCIRM129_08275	**270**	**CyDye**	Elongation factor Tu	Translation elongation	**tuf**	CYTO	43,600	-88,542	62	19
PFCIRM134_09515	**270**	**CyDye**	Elongation factor Tu	Translation elongation	**tuf**	CYTO	43,500	-10,599	11	2
PFCIRM139_03730	**270**	**CyDye**	Elongation factor Tu	Translation elongation	**tuf**	CYTO	43,600	-9,490	11	2
PFCIRM516_02330	**270**	**CyDye**	Elongation factor Tu	Translation elongation	**tuf**	CYTO	43,500	-79,735	50	15
PFCIRM1_05425	**270**	**CyDye**	Elongation factor Tu	Translation elongation	**tuf**	CYTO	43,600	-62,039	44	11
PFCIRM118_01835	**270**	**CyDye**	Elongation factor Tu	Translation elongation	**tuf**	CYTO	43,600	-8,050	7	2
PFCIRM138_08795	**270**	**CyDye**	Elongation factor Tu	Translation elongation	**tuf**	CYTO	43,600	-70,058	46	13
PFCIRM527_07270	**270**	**CyDye**	Elongation factor Tu	Translation elongation	**tuf**	CYTO	43,600	-68,054	38	13
PFCIRM118_01835	**270**	**Shaving**	Elongation factor Tu	Translation elongation	**tuf**	CYTO	43,600	-47,602	27	9
PFCIRM121_11145	**270**	**Shaving**	Elongation factor Tu	Translation elongation	**tuf**	CYTO	43,600	-11,573	12	3
PFCIRM129_08275	**270**	**Shaving**	Elongation factor Tu	Translation elongation	**tuf**	CYTO	43,600	-14,001	12	3
PFCIRM134_09515	**270**	**Shaving**	Elongation factor Tu	Translation elongation	**tuf**	CYTO	43,500	-38,859	24	8
PFCIRM139_03730	**270**	**Shaving**	Elongation factor Tu	Translation elongation	**tuf**	CYTO	43,600	-19,254	20	4
PFCIRM456_10195	**270**	**Shaving**	Elongation factor Tu	Translation elongation	**tuf**	CYTO	43,600	-28,877	24	6
PFCIRM516_02330	**270**	**Shaving**	Elongation factor Tu	Translation elongation	**tuf**	CYTO	43,500	-41,332	29	8
PFCIRM516_11390	**315**	**CyDye**	dolichyl-phosphate beta-D-mannosyltransferase	Cell wall	**dpm**	CYTO	31,600	-15,678	16	4
PFCIRM1_05675	**315**	**CyDye**	dolichyl-phosphate beta-D-mannosyltransferase	Cell wall	**dpm**	CYTO	31,600	-44,022	38	9
PFCIRM118_00230	**315**	**CyDye**	dolichyl-phosphate beta-D-mannosyltransferase	Cell wall	**dpm**	CYTO	32,000	-35,647	35	8
PFCIRM138_03565	**315**	**CyDye**	dolichyl-phosphate beta-D-mannosyltransferase	Cell wall	**dpm**	CYTO	32,000	-25,936	28	7
PFCIRM527_01975	**315**	**CyDye**	dolichyl-phosphate beta-D-mannosyltransferase	Cell wall	**dpm**	CYTO	32,000	-23,717	25	7
PFCIRM1_05685	**318**	**Shaving**	Hypothetical protein	Pouf, without similarity to other proteins	**0**	CYTO	57,300	-4,333	11	2
PFCIRM516_02670	**355**	**CyDye**	Transaldolase 1	Main glycolytic pathways	**tal1**	CYTO	37,900	-5,222	7	2
PFCIRM456_09960	**376**	**CyDye**	NADH-quinone oxidoreductase chain C (NADH dehydrogenase I, chain C)	Membrane bioenergetics	**nuoC**	CYTO	50,200	-44,253	31	10
PFCIRM118_09360	**376**	**CyDye**	NADH-quinone oxidoreductase chain D (NADH dehydrogenase I, chain D)	Membrane bioenergetics	**nuoD**	CYTO	50,200	-15,724	10	4
PFCIRM134_09635	**398**	**Shaving**	Peroxiredoxin/Alkyl hydroperoxide reductase subunit C Thioredoxin peroxidase	Detoxification	**ahpC**	CYTO	20,900	-15,260	21	2
PFCIRM139_03610	**398**	**Shaving**	Peroxiredoxin/Alkyl hydroperoxide reductase subunit C Thioredoxin peroxidase	Detoxification	**ahpC**	CYTO	20,900	-16,711	30	3

Locus Tag	Cluster	Method	Description	Function	Gene name	SurfG+ Predicted Localization	Molecular Weight (Da)	log(E value)	Coverage (%)	Number Of Unique Peptides
PFCIRM1_06355	**442**	**Shaving**	Protein-export membrane protein secD	Protein secretion	**secD**	PSE	59,800	-11,387	4	3
PFCIRM118_05035	**459**	**Shaving**	Enolase 1	Main glycolytic pathways	**eno1**	CYTO	45,800	-29,433	16	4
PFCIRM139_01570	**459**	**Shaving**	Enolase 1	Main glycolytic pathways	**eno1**	CYTO	45,900	-26,376	19	5
PFCIRM516_05380	**459**	**Shaving**	Enolase 1	Main glycolytic pathways	**eno1**	CYTO	45,800	-28,571	21	5
PFCIRM1_06660	**459**	**Shaving**	Enolase 1	Main glycolytic pathways	**eno1**	CYTO	45,800	-8,185	9	2
PFCIRM516_05390	**461**	**Shaving**	Hypothetical protein	Pouf similar to other proteins from the same organism	**0**	SECRETED	18,200	-21,477	42	5
PFCIRM1_06920	**508**	**Shaving**	Trypsin-like serine protease	Transport/binding of proteins/peptides	**htrA1**	CYTO	28,600	-13,577	9	2
PFCIRM121_06725	**519**	**Shaving**	secreted transglycosydase	Cell wall	**0**	PSE	19,900	-15,363	38	2
PFCIRM122_07625	**519**	**Shaving**	secreted transglycosydase	Cell wall	**0**	PSE	19,900	-28,663	47	5
PFCIRM129_11920	**519**	**Shaving**	secreted transglycosydase	Cell wall	**0**	PSE	20,000	-52,589	43	6
PFCIRM139_07535	**519**	**Shaving**	secreted transglycosydase	Cell wall	**0**	PSE	20,000	-38,304	49	5
PFCIRM456_03610	**519**	**Shaving**	secreted transglycosydase	Cell wall	**0**	PSE	19,900	-37,560	58	5
PFCIRM516_09370	**519**	**Shaving**	secreted transglycosydase	Cell wall	**0**	PSE	20,400	-19,186	47	4
PFCIRM527_08570	**519**	**Shaving**	secreted transglycosydase	Cell wall	**0**	PSE	NA	-8,000	NA	3
PFCIRM1_06990	**519**	**Shaving**	secreted transglycosydase	Cell wall	**0**	PSE	20,000	-49,295	55	6
PFCIRM118_10130	**521**	**CyDye**	Hypothetical protein	Pouf similar to proteins from other organisms	**0**	CYTO	27,700	-20,573	23	4
PFCIRM138_00535	**521**	**CyDye**	Hypothetical protein	Pouf similar to proteins from other organisms	**0**	CYTO	27,800	-15,895	23	4
PFCIRM121_06705	**523**	**Shaving**	Peptidase Subtilases	Metabolism of amino acids and related molecules	**vpr**	PSE	54,700	-17,420	14	4
PFCIRM122_07605	**523**	**Shaving**	Peptidase Subtilases	Metabolism of amino acids and related molecules	**vpr**	PSE	54,600	-18,241	12	4
PFCIRM134_08800	**523**	**Shaving**	Peptidase Subtilases	Metabolism of amino acids and related molecules	**vpr**	PSE	54,700	-7,298	5	2
PFCIRM139_07515	**523**	**Shaving**	Peptidase Subtilases	Metabolism of amino acids and related molecules	**vpr**	PSE	54,600	-20,926	14	4
PFCIRM456_03630	**523**	**Shaving**	Peptidase Subtilases	Metabolism of amino acids and related molecules	**vpr**	PSE	51,100	-14,727	10	3
PFCIRM514_08785	**523**	**Shaving**	Peptidase Subtilases	Metabolism of amino acids and related molecules	**vpr**	PSE	54,700	-16,170	9	3
PFCIRM516_09350	**523**	**Shaving**	Peptidase Subtilases	Metabolism of amino acids and related molecules	**vpr**	PSE	54,700	-19,811	12	4
PFCIRM527_08590	**523**	**Shaving**	Peptidase Subtilases	Metabolism of amino acids and related molecules	**vpr**	PSE	51,100	-16,825	10	3
PFCIRM1_07010	**523**	**Shaving**	Peptidase Subtilases	Metabolism of amino acids and related molecules	**vpr**	PSE	51,100	-22,839	14	5
PFCIRM139_08815	**536**	**Shaving**	Succinate dehydrogenase	Membrane bioenergetics	**sdhB3**	CYTO	27,000	-15,584	10	2
PFCIRM139_08820	**537**	**Shaving**	Succinate dehydrogenase flavoprotein subunit	Membrane bioenergetics	**sdhA3**	CYTO	74,700	-12,754	4	2
PFCIRM139_08850	**543**	**Shaving**	16S rRNA processing protein	Ribosomal proteins	**rimM**	CYTO	17,700	-12,402	28	3
PFCIRM129_10650	**637**	**Shaving**	Glucose-6-phosphate isomerase (Phosphohexose isomerase)	Main glycolytic pathways	**pgi**	CYTO	33,700	-10,656	13	2
PFCIRM514_09685	**637**	**Shaving**	Glucose-6-phosphate isomerase (Phosphohexose isomerase)	Main glycolytic pathways	**pgi**	CYTO	33,700	-6,177	11	2
PFCIRM1_10215	**637**	**Shaving**	Glucose-6-phosphate isomerase (Phosphohexose isomerase)	Main glycolytic pathways	**pgi**	CYTO	33,600	-12,818	13	2

Locus Tag	Cluster	Method	Description	Function	Gene name	SurfG+ Predicted Localization	Molecular Weight (Da)	log(E value)	Coverage (%)	Number Of Unique Peptides
PFCIRM139_08315	**656**	**CyDye**	2,5-diketo-D-gluconate reductase A	Metabolism of coenzymes and prosthetic groups	**dkgA**	CYTO	30,400	-13,669	20	4
PFCIRM121_10700	**672**	**Shaving**	peptidyl-prolyl cis-trans isomerase	Protein folding		SECRETED	35,800	-12,082	17	3
PFCIRM122_06285	**672**	**Shaving**	peptidyl-prolyl cis-trans isomerase	Protein folding		SECRETED	35,800	-10,835	20	4
PFCIRM129_09980	**672**	**Shaving**	peptidyl-prolyl cis-trans isomerase	Protein folding		SECRETED	35,900	-9,084	10	3
PFCIRM138_04965	**672**	**Shaving**	peptidyl-prolyl cis-trans isomerase	Protein folding		SECRETED	35,800	-6,878	5	2
PFCIRM139_00840	**672**	**Shaving**	peptidyl-prolyl cis-trans isomerase	Protein folding		SECRETED	35,900	-9,308	10	3
PFCIRM456_01975	**672**	**Shaving**	peptidyl-prolyl cis-trans isomerase	Protein folding		SECRETED	35,900	-11,379	13	3
PFCIRM514_02235	**672**	**Shaving**	peptidyl-prolyl cis-trans isomerase	Protein folding		SECRETED	35,900	-10,573	10	3
PFCIRM1_11270	**672**	**Shaving**	peptidyl-prolyl cis-trans isomerase	Protein folding		SECRETED	35,900	-9,482	15	3
PFCIRM118_07930	**681**	**Shaving**	CTP synthase (UTP--ammonia ligase) (CTP synthetase)	Metabolism of nucleotides and nucleic acids	**pyrG**	CYTO	63,700	-9,837	4	2
PFCIRM122_12040	**701**	**CyDye**	Elongation factor Ts (EF-Ts)	Translation elongation	**tsf**	CYTO	28,800	-16,979	9	3
PFCIRM129_11075	**701**	**CyDye**	Elongation factor Ts (EF-Ts)	Translation elongation	**tsf**	CYTO	28,800	-20,484	26	5
PFCIRM139_12390	**701**	**CyDye**	Elongation factor Ts (EF-Ts)	Translation elongation	**tsf**	CYTO	28,800	-28,109	26	7
PFCIRM456_08500	**701**	**CyDye**	Elongation factor Ts (EF-Ts)	Translation elongation	**tsf**	CYTO	28,800	-12,118	16	3
PFCIRM516_00855	**701**	**CyDye**	Elongation factor Ts (EF-Ts)	Translation elongation	**tsf**	CYTO	28,800	-37,116	40	8
PFCIRM1_11415	**701**	**CyDye**	Elongation factor Ts (EF-Ts)	Translation elongation	**tsf**	CYTO	28,800	-31,240	28	6
PFCIRM138_01405	**701**	**CyDye**	Elongation factor Ts (EF-Ts)	Translation elongation	**tsf**	CYTO	28,800	-31,200	20	5
PFCIRM527_01005	**701**	**CyDye**	Elongation factor Ts (EF-Ts)	Translation elongation	**tsf**	CYTO	28,800	-24,141	20	5
PFCIRM139_04860	**710**	**Shaving**	Hypothetical protein	Pouf similar to proteins from other organisms		PSE	10,700	-9,985	32	2
PFCIRM122_07445	**728**	**CyDye**	Pyruvate phosphate dikinase	Metabolism of carbohydrates And related molecules	**ppdk**	CYTO	95,700	-85,038	30	19
PFCIRM129_01500	**728**	**CyDye**	Pyruvate phosphate dikinase	Metabolism of carbohydrates And related molecules	**ppdk**	CYTO	95,700	-95,012	24	17
PFCIRM134_06645	**728**	**CyDye**	Pyruvate phosphate dikinase	Metabolism of carbohydrates And related molecules	**ppdk**	CYTO	95,700	-127,458	39	29
PFCIRM516_12725	**728**	**CyDye**	Pyruvate phosphate dikinase	Metabolism of carbohydrates And related molecules	**ppdk**	CYTO	95,700	-134,636	47	32
PFCIRM1_01390	**728**	**CyDye**	Pyruvate phosphate dikinase	Metabolism of carbohydrates And related molecules	**ppdk**	CYTO	95,700	-35,622	15	8
PFCIRM527_12240	**728**	**CyDye**	Pyruvate phosphate dikinase	Metabolism of carbohydrates And related molecules	**ppdk**	CYTO	95,700	-211,512	51	36
PFCIRM118_09205	**728**	**Shaving**	Pyruvate phosphate dikinase	Metabolism of carbohydrates And related molecules	**ppdk**	CYTO	95,700	-12,776	7	4

Locus Tag	Cluster	Method	Description	Function	Gene name	SurfG+ Predicted Localization	Molecular Weight (Da)	log(E value)	Coverage (%)	Number Of Unique Peptides
PFCIRM527_10710	**753**	**CyDye**	FAD linked oxidase domain protein	Membrane bioenergetics		CYTO	100,400	-58,541	17	13
PFCIRM118_05605	**754**	**CyDye**	Acyl-CoA dehydrogenase	Metabolism of lipids	**fad**	CYTO	40,800	-7,240	7	2
PFCIRM134_09020	**785**	**CyDye**	Chaperone protein dnaK 1 (Heat shock protein 70 1)	Protein folding	**dnaK1**	CYTO	65,300	-105,209	36	26
PFCIRM1_06910	**785**	**CyDye**	Chaperone protein dnaK 1 (Heat shock protein 70 1)	Protein folding	**dnaK1**	CYTO	65,300	-40,528	18	7
PFCIRM134_09020	**785**	**ClGua**	Chaperone protein dnaK 1 (Heat shock protein 70 1)	Protein folding	**dnaK1**	CYTO	65,300	-11,205	9	4
PFCIRM118_05275	**785**	**Shaving**	Chaperone protein dnaK 1 (Heat shock protein 70 1)	Protein folding	**dnaK1**	CYTO	65,300	-11,196	5	2
PFCIRM139_01305	**785**	**Shaving**	Chaperone protein dnaK 1 (Heat shock protein 70 1)	Protein folding	**dnaK1**	CYTO	65,300	-10,024	8	3
PFCIRM456_09350	**810**	**Shaving**	Hypothetical protein	Pouf, without similarity to other proteins		PSE	23,700	-8,369	23	3
PFCIRM516_10105	**860**	**Shaving**	binding protein of phosphate ABC transporter (BP:MOI:undef:phosphate)	Transport/binding of inorganic ions	**pstS**	PSE	36,400	-11,210	10	2
PFCIRM134_00955	**875**	**CyDye**	Aspartate-semialdehyde dehydrogenase (Semialdehyde dehydrogenase)	Metabolism of carbohydrates And related molecules	**asd**	CYTO	36,400	-29,176	28	7
PFCIRM1_07715	**875**	**CyDye**	Aspartate-semialdehyde dehydrogenase (Semialdehyde dehydrogenase)	Metabolism of carbohydrates And related molecules	**asd**	CYTO	36,500	-14,189	21	4
PFCIRM118_03210	**875**	**CyDye**	Aspartate-semialdehyde dehydrogenase (Semialdehyde dehydrogenase)	Metabolism of carbohydrates And related molecules	**asd**	CYTO	36,400	-50,233	36	9
PFCIRM138_08080	**875**	**CyDye**	Aspartate-semialdehyde dehydrogenase (Semialdehyde dehydrogenase)	Metabolism of carbohydrates And related molecules	**asd**	CYTO	36,400	-55,557	36	9
PFCIRM527_04750	**875**	**CyDye**	Aspartate-semialdehyde dehydrogenase (Semialdehyde dehydrogenase)	Metabolism of carbohydrates And related molecules	**asd**	CYTO	36,400	-52,201	39	9
PFCIRM134_01025	**889**	**CyDye**	chaperone clpC (ATP-dependent Clp protease ATP-binding subunit)	Protein folding	**clpC**	CYTO	NA	-58,238	NA	15
PFCIRM1_07785	**889**	**CyDye**	chaperone clpC (ATP-dependent Clp protease ATP-binding subunit)	Protein folding	**clpC**	CYTO	94,200	-136,850	36	25
PFCIRM118_03140	**889**	**CyDye**	chaperone clpC (ATP-dependent Clp protease ATP-binding subunit)	Protein folding	**clpC**	CYTO	94,200	-80,423	27	16
PFCIRM1_07785	**889**	**Shaving**	chaperone clpC (ATP-dependent Clp protease ATP-binding subunit)	Protein folding	**clpC**	CYTO	94,200	-8,357	3	2
PFCIRM122_12425	**917**	**CyDye**	Phenylalanyl-tRNA synthetase beta chain (Phenylalanine-- tRNA ligase beta chain)	Aminoacyl-tRNA synthetases	**pheT**	CYTO	89,900	-14,401	7	4
PFCIRM514_07125	**945**	**CyDye**	Hypothetical protein	Pouf, without similarity to other proteins		MEMBRANE	36,800	-5,678	8	2
PFCIRM516_04180	**945**	**CyDye**	Hypothetical protein	Pouf, without similarity to other proteins		MEMBRANE	36,800	-14,403	21	5
PFCIRM122_01005	**951**	**CyDye**	Methylmalonic acid semialdehyde dehydrogenase	Specific carbohydrate metabolic pathway	**iolA (msdA**	CYTO	52,700	-31,902	16	5
PFCIRM118_10555	**951**	**Shaving**	Methylmalonic acid semialdehyde dehydrogenase	Specific carbohydrate metabolic pathway	**iolA (msdA**	CYTO	52,700	-13,970	6	2
PFCIRM516_04260	**961**	**CyDye**	Lysine exporter protein, membran protein	Transport/binding of amino-acids	**lysE**	MEMBRANE	63,200	-22,900	19	8
PFCIRM1_09590	**961**	**CyDye**	Lysine exporter protein, membran protein	Transport/binding of amino-acids	**ilvD**	CYTO	64,900	-67,434	25	11
PFCIRM118_10500	**961**	**CyDye**	Dihydroxy-acid dehydratase	Metabolism of carbohydrates And related molecules	**ilvD**	CYTO	64,900	-39,924	18	8
PFCIRM118_10440	**974**	**Shaving**	Methylmalonyl-CoA carboxytransferase 5S subunit. (transcarboxylase 5S) 505 bp	Specific carbohydrate metabolic pathway		CYTO	55,500	-16,351	6	2
PFCIRM516_04330	**975**	**CyDye**	Methylmalonyl-CoA carboxytransferase 12S subunit (Transcarboxylase 12S subunit)	Specific carbohydrate metabolic pathway	**mmdA**	CYTO	56,300	-79,818	42	18
PFCIRM118_10430	**975**	**Shaving**	Methylmalonyl-CoA carboxytransferase 12S subunit (Transcarboxylase 12S subunit)	Specific carbohydrate metabolic pathway	**mmdA**	CYTO	56,300	-13,818	5	2
PFCIRM118_10420	**977**	**Shaving**	Methylmalonyl-CoA carboxytransferase (Transcarboxylase, 1.3S subunit)	Specific carbohydrate metabolic pathway	**bccp**	CYTO	12,300	-23,612	39	3
PFCIRM527_09640	**980**	**CyDye**	phosphoglycerate mutase	Main glycolytic pathways	**gpm2**	CYTO	27,800	-19,369	30	5

Locus Tag	Cluster	Method	Description	Function	Gene name	SurfG+ Predicted Localization	Molecular Weight (Da)	log(E value)	Coverage (%)	Number Of Unique Peptides
PFCIRM122_06000	**1043**	**CyDye**	Heat shock protein 20 2 (20 kDa chaperone 2)	Protein folding	**hsp20 2**	CYTO	16,800	-29,445	64	7
PFCIRM129_03120	**1043**	**CyDye**	Heat shock protein 20 2 (20 kDa chaperone 2)	Protein folding	**hsp20 2**	CYTO	16,800	-24,827	51	6
PFCIRM134_10890	**1043**	**CyDye**	Heat shock protein 20 2 (20 kDa chaperone 2)	Protein folding	**hsp20 2**	CYTO	16,800	-23,715	44	5
PFCIRM139_07050	**1043**	**CyDye**	Heat shock protein 20 2 (20 kDa chaperone 2)	Protein folding	**hsp20 2**	CYTO	16,800	-25,979	53	5
PFCIRM456_00640	**1043**	**CyDye**	Heat shock protein 20 2 (20 kDa chaperone 2)	Protein folding	**hsp20 2**	CYTO	16,800	-34,842	64	8
PFCIRM516_08345	**1043**	**CyDye**	Heat shock protein 20 2 (20 kDa chaperone 2)	Protein folding	**hsp20 2**	CYTO	16,800	-30,305	59	6
PFCIRM1_10645	**1043**	**CyDye**	Heat shock protein 20 2 (20 kDa chaperone 2)	Protein folding	**hsp20 2**	CYTO	16,800	-39,412	58	6
PFCIRM118_09610	**1043**	**CyDye**	Heat shock protein 20 2 (20 kDa chaperone 2)	Protein folding	**hsp20 2**	CYTO	16,800	-23,223	38	4
PFCIRM138_10010	**1043**	**CyDye**	Heat shock protein 20 2 (20 kDa chaperone 2)	Protein folding	**hsp20 2**	CYTO	16,800	-36,855	59	6
PFCIRM527_10125	**1043**	**CyDye**	Heat shock protein 20 2 (20 kDa chaperone 2)	Protein folding	**hsp20 2**	CYTO	16,800	-35,753	53	5
PFCIRM118_09610	**1043**	**Shaving**	Heat shock protein 20 2 (20 kDa chaperone 2)	Protein folding	**hsp20 2**	CYTO	16,800	-23,203	31	3
PFCIRM516_08345	**1043**	**Shaving**	Heat shock protein 20 2 (20 kDa chaperone 2)	Protein folding	**hsp20 2**	CYTO	16,800	-23,975	40	3
PFCIRM1_10645	**1043**	**Shaving**	Heat shock protein 20 2 (20 kDa chaperone 2)	Protein folding	**hsp20 2**	CYTO	16,800	-17,702	25	2
PFCIRM122_05975	**1047**	**CyDye**	4-aminobutyrate aminotransferase	Metabolism of carbohydrates And related molecules	**gabT**	CYTO	46,600	-44,590	25	11
PFCIRM456_00680	**1050**	**CyDye**	Peptide chain release factor 2 (RF-2)	Nonribosomal protein synthesis	**prfB**	CYTO	41,000	-26,360	25	5
PFCIRM1_10700	**1053**	**Shaving**	Hypothetical protein	Pouf, without similarity to other proteins		CYTO	48,000	-8,173	6	2
PFCIRM1_10740	**1061**	**CyDye**	Preprotein translocase SecA subunit	Transport/binding proteins and lipoproteins		CYTO	106,300	-61,144	22	15
PFCIRM138_10105	**1061**	**CyDye**	Preprotein translocase SecA subunit	Transport/binding proteins and lipoproteins		CYTO	106,300	-62,053	21	13
PFCIRM139_06890	**1072**	**Shaving**	Cold shock-like protein CspA	Adaptation to atypical conditions	**cspA**	CYTO	7,300	-10,012	24	2
PFCIRM456_04270	**1094**	**CyDye**	binding protein of oligopeptide ABC transporter	Transport/binding of amino-acids		PSE	54,900	-9,945	9	3
PFCIRM134_11390	**1134**	**CyDye**	ATP synthase subunit alpha (ATP synthase F1 sector subunit alpha)	Membrane bioenergetics	**atpA**	CYTO	58,800	-51,207	28	13
PFCIRM138_10500	**1134**	**CyDye**	ATP synthase subunit alpha (ATP synthase F1 sector subunit alpha)	Membrane bioenergetics	**atpA**	CYTO	58,900	-36,040	25	10
PFCIRM456_04495	**1136**	**CyDye**	ATP synthase subunit beta (ATP synthase F1 sector subunit beta)	Membrane bioenergetics	**atpD**	CYTO	52,400	-92,352	64	21
PFCIRM527_08960	**1136**	**CyDye**	ATP synthase subunit beta (ATP synthase F1 sector subunit beta)	Membrane bioenergetics	**atpD**	CYTO	50,400	-97,438	63	21
PFCIRM134_11440	**1144**	**CyDye**	Hypothetical protein	Pouf similar to proteins from other organisms		CYTO	63,900	-4,208	3	2
PFCIRM516_09000	**1144**	**CyDye**	Hypothetical protein	Pouf similar to proteins from other organisms		CYTO	63,900	-18,313	14	6
PFCIRM129_05085	**1148**	**CyDye**	Phosphoglucomutase	Specific carbohydrate metabolic pathway	**pgm1**	CYTO	58,600	-5,819	5	2
PFCIRM118_02025	**1148**	**CyDye**	Phosphoglucomutase	Specific carbohydrate metabolic pathway	**pgm1**	CYTO	58,600	-5,422	4	2
PFCIRM118_11145	**1168**	**Shaving**	serine protease, trypsin-like serine proteases	Transport/binding of proteins/peptides	**htrA2**	PSE	47,700	-13,666	7	2
PFCIRM121_07190	**1168**	**Shaving**	serine protease, trypsin-like serine proteases	Transport/binding of proteins/peptides	**htrA2**	PSE	47,700	-17,283	10	3
PFCIRM122_09940	**1168**	**Shaving**	serine protease, trypsin-like serine proteases	Transport/binding of proteins/peptides	**htrA2**	PSE	47,700	-9,945	7	2
PFCIRM516_11155	**1168**	**Shaving**	serine protease, trypsin-like serine proteases	Transport/binding of proteins/peptides	**htrA2**	PSE	47,800	-13,217	10	3
PFCIRM1_00060	**1168**	**Shaving**	serine protease, trypsin-like serine proteases	Transport/binding of proteins/peptides	**htrA2**	PSE	47,700	-9,549	12	3
PFCIRM1_00345	**1184**	**Shaving**	Ferrous iron uptake protein B 9.A.8.1.x	Transport/binding of inorganic ions	**feoB**	MEMBRANE	66,300	-12,000	5	2
PFCIRM134_12030	**1204**	**CyDye**	Galactose-1-phosphate uridylyltransferase	Specific carbohydrate metabolic pathway	**galT**	CYTO	75,100	-5,207	3	2
PFCIRM134_12110	**1223**	**ClGua**	Trigger factor (TF)	Protein folding	**tig**	CYTO	57,300	-5,740	5	2
PFCIRM118_11995	**1223**	**Shaving**	Trigger factor (TF)	Protein folding	**tig**	CYTO	57,300	-14,049	7	2
PFCIRM129_09060	**1238**	**Shaving**	Hypothetical protein	Pouf, without similarity to other proteins		CYTO	26,100	-9,913	13	2

Locus Tag	Cluster	Method	Description	Function	Gene name	SurfG+ Predicted Localization	Molecular Weight (Da)	log(E value)	Coverage (%)	Number Of Unique Peptides
PFCIRM121_03270	**1239**	**Shaving**	Hypothetical secreted protein	Pouf similar to other proteins from the same organism		PSE	26,200	-9,278	22	3
PFCIRM122_02285	**1239**	**Shaving**	Hypothetical secreted protein	Pouf similar to other proteins from the same organism		PSE	26,200	-16,358	26	4
PFCIRM134_06455	**1239**	**Shaving**	Hypothetical secreted protein	Pouf similar to other proteins from the same organism		PSE	26,100	-6,316	13	2
PFCIRM456_06035	**1239**	**Shaving**	Hypothetical secreted protein	Pouf similar to other proteins from the same organism		PSE	26,200	-11,996	30	5
PFCIRM516_12900	**1239**	**Shaving**	Hypothetical secreted protein	Pouf similar to other proteins from the same organism		PSE	26,200	-9,346	13	2
PFCIRM1_01335	**1239**	**Shaving**	Hypothetical secreted protein	Pouf similar to other proteins from the same organism		PSE	26,200	-9,305	17	2
PFCIRM139_08410	**1258**	**Shaving**	50S ribosomal protein L9	Ribosomal proteins	**rplI**	CYTO	16,000	-9,711	25	2
PFCIRM122_06630	**1266**	**Shaving**	polar amino acid ABC transporter, Binding protein component	Transport/binding of amino-acids		PSE	30,000	-17,271	30	5
PFCIRM516_02085	**1266**	**Shaving**	polar amino acid ABC transporter, Binding protein component	Transport/binding of amino-acids		PSE	30,000	-26,832	26	5
PFCIRM129_03750	**1305**	**CyDye**	Phosphate acetyltransferase	Metabolism of carbohydrates And related molecules	**pta**	CYTO	52,800	-41,322	27	10
PFCIRM456_04975	**1305**	**CyDye**	Phosphate acetyltransferase	Metabolism of carbohydrates And related molecules	**pta**	CYTO	52,700	-29,589	25	8
PFCIRM118_06510	**1310**	**Shaving**	30S ribosomal protein S10	Ribosomal proteins	**rpsJ**	CYTO	24,700	-13,642	13	2
PFCIRM118_06520	**1314**	**Shaving**	50S ribosomal protein L2	Ribosomal proteins	**rplB**	CYTO	30,300	-6,636	11	2
PFCIRM1_03420	**1316**	**Shaving**	50S ribosomal protein L22	Ribosomal proteins	**rplV**	CYTO	29,900	-10,882	12	2
PFCIRM118_06620	**1335**	**Shaving**	solute binding protein of the ABC transport system	Transport/binding of proteins/peptides	**bopA**	PSE	61,400	-38,058	27	13
PFCIRM129_08120	**1335**	**Shaving**	solute binding protein of the ABC transport system	Transport/binding of proteins/peptides	**bopA**	PSE	61,400	-60,005	33	13
PFCIRM134_05300	**1335**	**Shaving**	solute binding protein of the ABC transport system	Transport/binding of proteins/peptides	**bopA**	PSE	61,300	-94,480	39	26
PFCIRM139_03885	**1335**	**Shaving**	solute binding protein of the ABC transport system	Transport/binding of proteins/peptides	**bopA**	PSE	61,400	-15,126	6	2
PFCIRM456_10340	**1335**	**Shaving**	solute binding protein of the ABC transport system	Transport/binding of proteins/peptides	**bopA**	PSE	61,600	-17,081	10	4
PFCIRM514_01120	**1335**	**Shaving**	solute binding protein of the ABC transport system	Transport/binding of proteins/peptides	**bopA**	PSE	61,400	-172,578	50	42
PFCIRM516_06415	**1335**	**Shaving**	solute binding protein of the ABC transport system	Transport/binding of proteins/peptides	**bopA**	PSE	61,700	-70,449	34	22
PFCIRM1_03510	**1335**	**Shaving**	solute binding protein of the ABC transport system	Transport/binding of proteins/peptides	**bopA**	PSE	61,400	-123,828	46	27
PFCIRM1_03580	**1349**	**Shaving**	30S ribosomal protein S4	Ribosomal proteins	**rpsD**	CYTO	23,200	-4,564	10	2
PFCIRM456_10415	**1350**	**CyDye**	DNA-directed RNA polymerase alpha chain (RNA polymerase subunit alpha)	Transcription elongation	**rpoA**	CYTO	36,900	-51,606	41	12
PFCIRM139_03980	**1354**	**CyDye**	Resuscitation-promoting factor RpfB	Adaptation to atypical conditions		SECRETED	NA	-27,491	NA	7
PFCIRM118_06715	**1354**	**Shaving**	Resuscitation-promoting factor RpfB	Adaptation to atypical conditions		SECRETED	37,700	-12,983	8	2
PFCIRM121_06075	**1354**	**Shaving**	Resuscitation-promoting factor RpfB	Adaptation to atypical conditions		SECRETED	37,600	-56,105	41	9
PFCIRM122_08000	**1354**	**Shaving**	Resuscitation-promoting factor RpfB	Adaptation to atypical conditions		SECRETED	37,600	-39,740	36	9
PFCIRM129_08025	**1354**	**Shaving**	Resuscitation-promoting factor RpfB	Adaptation to atypical conditions		SECRETED	37,700	-46,300	36	9
PFCIRM134_05395	**1354**	**Shaving**	Resuscitation-promoting factor RpfB	Adaptation to atypical conditions		SECRETED	37,700	-32,524	32	7
PFCIRM139_03980	**1354**	**Shaving**	Resuscitation-promoting factor RpfB	Adaptation to atypical conditions		SECRETED	37,600	-47,287	36	9
PFCIRM456_10430	**1354**	**Shaving**	Resuscitation-promoting factor RpfB	Adaptation to atypical conditions		SECRETED	37,600	-39,223	36	9
PFCIRM514_01030	**1354**	**Shaving**	Resuscitation-promoting factor RpfB	Adaptation to atypical conditions		SECRETED	37,600	-42,999	36	9
PFCIRM516_06320	**1354**	**Shaving**	Resuscitation-promoting factor RpfB	Adaptation to atypical conditions		SECRETED	37,600	-42,137	29	7
PFCIRM527_07030	**1354**	**Shaving**	Resuscitation-promoting factor RpfB	Adaptation to atypical conditions		SECRETED	37,600	-37,203	36	9
PFCIRM1_03605	**1354**	**Shaving**	Resuscitation-promoting factor RpfB	Adaptation to atypical conditions		SECRETED	37,700	-41,741	32	7

Locus Tag	Cluster	Method	Description	Function	Gene name	SurfG+ Predicted Localization	Molecular Weight (Da)	log(E value)	Coverage (%)	Number Of Unique Peptides
PFCIRM122_08005	**1355**	**CyDye**	Iron/Manganese superoxide dismutase (Superoxide dismutase [Mn/Fe])	Detoxification	**sodA**	CYTO	22,700	-14,417	12	4
PFCIRM139_03985	**1355**	**CyDye**	Iron/Manganese superoxide dismutase (Superoxide dismutase [Mn/Fe])	Detoxification	**sodA**	CYTO	22,700	-18,974	14	5
PFCIRM138_09035	**1355**	**CyDye**	Iron/Manganese superoxide dismutase (Superoxide dismutase [Mn/Fe])	Detoxification	**sodA**	CYTO	22,700	-6,650	11	2
PFCIRM129_07955	**1367**	**CyDye**	GTP phosphohydrolase (mRNA-translation-assisting)	Translation elongation		CYTO	75,000	-29,102	15	7
PFCIRM118_09520	**1388**	**Shaving**	cell-wall peptidases, NlpC/P60 family secreted protein	Cell wall		SECRETED	59,500	-25,207	12	4
PFCIRM121_09150	**1388**	**Shaving**	cell-wall peptidases, NlpC/P60 family secreted protein	Cell wall		SECRETED	58,700	-98,081	51	19
PFCIRM122_07115	**1388**	**Shaving**	cell-wall peptidases, NlpC/P60 family secreted protein	Cell wall		SECRETED	58,700	-62,587	38	14
PFCIRM129_08670	**1388**	**Shaving**	cell-wall peptidases, NlpC/P60 family secreted protein	Cell wall		SECRETED	58,700	-57,823	36	12
PFCIRM134_09930	**1388**	**Shaving**	cell-wall peptidases, NlpC/P60 family secreted protein	Cell wall		SECRETED	58,700	-46,394	23	8
PFCIRM138_02375	**1388**	**Shaving**	cell-wall peptidases, NlpC/P60 family secreted protein	Cell wall		SECRETED	58,700	-17,509	3	2
PFCIRM139_03330	**1388**	**Shaving**	cell-wall peptidases, NlpC/P60 family secreted protein	Cell wall		SECRETED	59,500	-68,171	44	16
PFCIRM456_09805	**1388**	**Shaving**	cell-wall peptidases, NlpC/P60 family secreted protein	Cell wall		SECRETED	59,600	-105,230	48	18
PFCIRM514_01290	**1388**	**Shaving**	cell-wall peptidases, NlpC/P60 family secreted protein	Cell wall		SECRETED	59,500	-84,058	44	16
PFCIRM516_02730	**1388**	**Shaving**	cell-wall peptidases, NlpC/P60 family secreted protein	Cell wall		SECRETED	59,500	-65,152	36	13
PFCIRM527_10435	**1388**	**Shaving**	cell-wall peptidases, NlpC/P60 family secreted protein	Cell wall		SECRETED	58,700	-69,221	41	15
PFCIRM1_08110	**1388**	**Shaving**	cell-wall peptidases, NlpC/P60 family secreted protein	Cell wall		SECRETED	58,700	-85,786	43	15
PFCIRM121_02000	**1395**	**Shaving**	Anaerobic dimethyl sulfoxide reductase chain C	Membrane bioenergetics	**dmsC**	MEMBRANE	23,400	-8,075	10	2
PFCIRM122_03750	**1395**	**Shaving**	Anaerobic dimethyl sulfoxide reductase chain C	Membrane bioenergetics	**dmsC**	MEMBRANE	23,400	-8,523	15	2
PFCIRM456_09190	**1395**	**Shaving**	Anaerobic dimethyl sulfoxide reductase chain C	Membrane bioenergetics	**dmsC**	MEMBRANE	23,400	-8,433	15	3
PFCIRM121_01995	**1396**	**Shaving**	Anaerobic dimethyl sulfoxide reductase chain A	Membrane bioenergetics	**dmsA**	CYTO	95,700	-28,759	9	5
PFCIRM122_03755	**1396**	**Shaving**	Anaerobic dimethyl sulfoxide reductase chain A	Membrane bioenergetics	**dmsA**	CYTO	96,900	-21,657	5	4
PFCIRM139_00505	**1396**	**Shaving**	Anaerobic dimethyl sulfoxide reductase chain A	Membrane bioenergetics	**dmsA**	CYTO	96,900	-14,338	4	3
PFCIRM456_09185	**1396**	**Shaving**	Anaerobic dimethyl sulfoxide reductase chain A	Membrane bioenergetics	**dmsA**	CYTO	96,300	-27,746	8	5
PFCIRM129_06835	**1411**	**Shaving**	50S ribosomal protein L21	Ribosomal proteins	**rplU**	CYTO	9,200	-4,286	30	2
PFCIRM1_08260	**1413**	**CyDye**	GTPase	Miscellaneous	**obg**	CYTO	57,200	-21,852	15	5
PFCIRM118_11870	**1413**	**CyDye**	GTPase	Miscellaneous	**obg**	CYTO	57,200	-6,213	5	2
PFCIRM516_07805	**1416**	**CyDye**	Gamma-glutamyl phosphate reductase (Glutamate-5- semialdehyde dehydrogenase)	Metabolism of carbohydrates And related molecules	**proA**	CYTO	NA	-5,062	NA	3
PFCIRM134_07290	**1467**	**CyDye**	Aspartyl-tRNA synthetase (Aspartate--tRNA ligase) (AspRS)	.Aminoacyl-tRNA synthetases	**aspS**	CYTO	67,200	-33,092	13	7
PFCIRM456_02495	**1472**	**Shaving**	Glycine cleavage system T protein, Aminomethyltransferase	Metabolism of amino acids And related molecules	**gcvT1**	CYTO	43,700	-6,827	9	2
PFCIRM514_10565	**1472**	**Shaving**	Glycine cleavage system T protein, Aminomethyltransferase	Metabolism of amino acids And related molecules	**gcvT1**	CYTO	43,700	-10,840	9	3
PFCIRM514_00620	**1483**	**Shaving**	Hypothetical secreted protein	Pouf similar to proteins from other organisms		PSE	35,100	-16,917	10	2
PFCIRM527_02215	**1483**	**Shaving**	Hypothetical secreted protein	Pouf similar to proteins from other organisms		PSE	35,100	-7,511	8	2
PFCIRM1_08775	**1483**	**Shaving**	Hypothetical secreted protein	Pouf similar to proteins from other organisms		PSE	35,100	-12,222	16	2

Locus Tag	Cluster	Method	Description	Function	Gene name	SurfG+ Predicted Localization	Molecular Weight (Da)	log(E value)	Coverage (%)	Number Of Unique Peptides
PFCIRM122_08375	**1489**	**CyDye**	AzlC family protein precursor (amino acid permease)	Transport/binding of amino-acids	**azlC**	MEMBRANE	34,800	-23,296	26	7
PFCIRM129_07645	**1489**	**CyDye**	AzlC family protein precursor (amino acid permease)	Transport/binding of amino-acids	**azlC**	MEMBRANE	34,800	-8,337	17	3
PFCIRM134_05770	**1489**	**CyDye**	AzlC family protein precursor (amino acid permease)	Transport/binding of amino-acids	**azlC**	MEMBRANE	34,800	-27,976	29	10
PFCIRM139_04370	**1489**	**CyDye**	AzlC family protein precursor (amino acid permease)	Transport/binding of amino-acids	**azlC**	MEMBRANE	34,800	-28,841	30	10
PFCIRM514_00650	**1489**	**CyDye**	AzlC family protein precursor (amino acid permease)	Transport/binding of amino-acids	**azlC**	MEMBRANE	34,800	-34,627	39	11
PFCIRM1_08810	**1489**	**CyDye**	AzlC family protein precursor (amino acid permease)	Transport/binding of amino-acids	**mdh**	CYTO	34,800	-5,263	9	2
PFCIRM118_07085	**1489**	**CyDye**	Malate dehydrogenase	Metabolism of carbohydrates And related molecules	**mdh**	CYTO	34,800	-35,748	28	8
PFCIRM138_11215	**1489**	**CyDye**	Malate dehydrogenase	Metabolism of carbohydrates And related molecules	**mdh**	CYTO	34,800	-36,223	27	8
PFCIRM527_02175	**1489**	**CyDye**	Malate dehydrogenase	Metabolism of carbohydrates And related molecules	**mdh**	CYTO	34,800	-31,324	27	8
PFCIRM118_07085	**1489**	**Shaving**	AzlC family protein precursor (amino acid permease)	Transport/binding of amino-acids	**azlC**	MEMBRANE	34,800	-7,126	9	2
PFCIRM139_04370	**1489**	**Shaving**	AzlC family protein precursor (amino acid permease)	Transport/binding of amino-acids	**azlC**	MEMBRANE	34,800	-8,234	9	2
PFCIRM516_05945	**1489**	**Shaving**	AzlC family protein precursor (amino acid permease)	Transport/binding of amino-acids	**azlC**	MEMBRANE	34,800	-4,796	9	2
PFCIRM122_00770	**1506**	**CyDye**	Hypothetical protein	Pouf similar to proteins from other organisms		CYTO	23,100	-11,873	26	3
PFCIRM134_04800	**1506**	**CyDye**	Hypothetical protein	Pouf similar to proteins from other organisms		CYTO	23,100	-14,205	33	5
PFCIRM139_11245	**1506**	**CyDye**	Hypothetical protein	Pouf similar to proteins from other organisms		CYTO	23,100	-12,571	23	4
PFCIRM456_11770	**1506**	**CyDye**	Hypothetical protein	Pouf similar to proteins from other organisms		CYTO	23,100	-8,883	17	3
PFCIRM139_11245	**1506**	**Shaving**	Hypothetical protein	Pouf similar to proteins from other organisms		CYTO	23,100	-9,597	26	4
PFCIRM134_04805	**1507**	**Shaving**	iron-sulfur protein	Membrane bioenergetics		CYTO	57,200	-18,648	8	3
PFCIRM118_01135	**1515**	**Shaving**	Hypothetical protein	Pouf, without similarity to other proteins		PSE	24,400	-25,364	26	3
PFCIRM134_12655	**1515**	**Shaving**	Hypothetical protein	Pouf, without similarity to other proteins		PSE	24,500	-20,986	22	3
PFCIRM514_08110	**1515**	**Shaving**	Hypothetical protein	Pouf, without similarity to other proteins		PSE	24,400	-10,890	9	3
PFCIRM1_08955	**1515**	**Shaving**	Hypothetical protein	Pouf, without similarity to other proteins		PSE	24,100	-18,621	26	4
PFCIRM121_10030	**1553**	**Shaving**	penicillin-binding protein (peptidoglycan glycosyltransferase)	Cell wall	**mrc/ponA**	PSE	75,300	-41,584	19	9
PFCIRM122_08625	**1553**	**Shaving**	penicillin-binding protein (peptidoglycan glycosyltransferase)	Cell wall	**mrc/ponA**	PSE	77,600	-44,185	18	8
PFCIRM134_02320	**1553**	**Shaving**	penicillin-binding protein (peptidoglycan glycosyltransferase)	Cell wall	**mrc/ponA**	PSE	77,500	-16,246	11	3
PFCIRM456_11945	**1553**	**Shaving**	penicillin-binding protein (peptidoglycan glycosyltransferase)	Cell wall	**mrc/ponA**	PSE	77,500	-29,316	16	7
PFCIRM514_06620	**1553**	**Shaving**	penicillin-binding protein (peptidoglycan glycosyltransferase)	Cell wall	**mrc/ponA**	PSE	77,600	-53,223	21	10
PFCIRM516_12415	**1553**	**Shaving**	penicillin-binding protein (peptidoglycan glycosyltransferase)	Cell wall	**mrc/ponA**	PSE	75,300	-52,637	28	12
PFCIRM527_06860	**1553**	**Shaving**	penicillin-binding protein (peptidoglycan glycosyltransferase)	Cell wall	**mrc/ponA**	PSE	75,300	-20,748	11	4
PFCIRM1_09170	**1553**	**Shaving**	penicillin-binding protein (peptidoglycan glycosyltransferase)	Cell wall	**mrc/ponA**	PSE	75,200	-39,842	19	8

Locus Tag	Cluster	Method	Description	Function	Gene name	SurfG+ Predicted Localization	Molecular Weight (Da)	log(E value)	Coverage (%)	Number Of Unique Peptides
PFCIRM122_11195	**1599**	**CyDye**	Membrane protease subunits, Stomatin/prohibitin homologs	Protein degradation		MEMBRANE	33,500	-15,525	28	4
PFCIRM134_01600	**1599**	**CyDye**	Membrane protease subunits, Stomatin/prohibitin homologs	Protein degradation		MEMBRANE	33,500	-15,011	22	3
PFCIRM139_11500	**1599**	**CyDye**	Membrane protease subunits, Stomatin/prohibitin homologs	Protein degradation		MEMBRANE	NA	-80,100	NA	18
PFCIRM514_01860	**1599**	**CyDye**	Membrane protease subunits, Stomatin/prohibitin homologs	Protein degradation		MEMBRANE	33,500	-129,461	69	25
PFCIRM516_04965	**1599**	**CyDye**	Membrane protease subunits, Stomatin/prohibitin homologs	Protein degradation		MEMBRANE	33,500	-23,600	37	5
PFCIRM1_09420	**1599**	**CyDye**	Membrane protease subunits, Stomatin/prohibitin homologs	Protein degradation	**cys2**	CYTO	33,500	-76,304	64	13
PFCIRM118_04615	**1599**	**CyDye**	cysteine synthase 2	Metabolism of carbohydrates And related molecules	**cys2**	CYTO	33,500	-86,796	60	16
PFCIRM138_09490	**1599**	**CyDye**	cysteine synthase 2	Metabolism of carbohydrates And related molecules	**cys2**	CYTO	33,500	-93,505	68	15
PFCIRM527_00170	**1599**	**CyDye**	cysteine synthase 2	Metabolism of carbohydrates And related molecules	**cys2**	CYTO	33,500	-99,643	68	15
PFCIRM134_01600	**1599**	**ClGua**	Membrane protease subunits, Stomatin/prohibitin homologs	Protein degradation		MEMBRANE	33,500	-36,541	37	8
PFCIRM118_01345	**1613**	**Shaving**	Hypothetical protein	Pouf, without similarity to other proteins		PSE	29,900	-31,809	26	5
PFCIRM121_03250	**1613**	**Shaving**	Hypothetical protein	Pouf, without similarity to other proteins		PSE	29,900	-12,241	24	3
PFCIRM134_02745	**1613**	**Shaving**	Hypothetical protein	Pouf, without similarity to other proteins		PSE	29,900	-16,282	15	3
PFCIRM139_10165	**1613**	**Shaving**	Hypothetical protein	Pouf, without similarity to other proteins		PSE	30,000	-10,055	15	2
PFCIRM456_05235	**1613**	**Shaving**	Hypothetical protein	Pouf, without similarity to other proteins		PSE	29,900	-21,560	24	4
PFCIRM514_08320	**1613**	**Shaving**	Hypothetical protein	Pouf, without similarity to other proteins		PSE	30,000	-17,182	12	2
PFCIRM516_01875	**1613**	**Shaving**	Hypothetical protein	Pouf, without similarity to other proteins		PSE	29,900	-18,679	20	3
PFCIRM527_06050	**1613**	**Shaving**	Hypothetical protein	Pouf, without similarity to other proteins		PSE	29,900	-27,164	29	5
PFCIRM1_09755	**1613**	**Shaving**	Hypothetical protein	Pouf, without similarity to other proteins		PSE	30,000	-19,171	20	3
PFCIRM1_09895	**1640**	**CyDye**	Phosphoketolase pyrophosphate	Main glycolytic pathways	**phk**	CYTO	96,600	-37,005	12	8
PFCIRM138_02615	**1640**	**CyDye**	Phosphoketolase pyrophosphate	Main glycolytic pathways	**phk**	CYTO	96,600	-22,711	8	6
PFCIRM527_11260	**1640**	**CyDye**	Phosphoketolase pyrophosphate	Main glycolytic pathways	**phk**	CYTO	96,600	-19,550	8	6
PFCIRM139_06195	**1644**	**Shaving**	Anaerobic glycerol-3-phosphate dehydrogenase Subunit C	Specific carbohydrate metabolic pathway	**glpC**	CYTO	50,500	-9,730	5	2
PFCIRM134_00210	**1645**	**CyDye**	Anaerobic glycerol-3-phosphate dehydrogenase Subunit B	Specific carbohydrate metabolic pathway	**glpB**	CYTO	45,800	-51,329	42	13
PFCIRM516_02950	**1645**	**CyDye**	Anaerobic glycerol-3-phosphate dehydrogenase Subunit B	Specific carbohydrate metabolic pathway	**glpB**	CYTO	45,800	-44,521	36	10
PFCIRM139_07805	**1689**	**CyDye**	DNA polymerase III, beta chain	DNA replication	**dnaN**	CYTO	41,400	-25,866	21	8
PFCIRM129_05460	**1692**	**CyDye**	surface layer protein E (S-layer protein E)	Cell wall	**slpE**	PSE	59,200	-8,677	4	2
PFCIRM129_05460	**1692**	**ClGua**	surface layer protein E (S-layer protein E)	Cell wall	**slpE**	PSE	59,200	-35,405	15	6
PFCIRM129_05460	**1692**	**Shaving**	surface layer protein E (S-layer protein E)	Cell wall	**slpE**	PSE	59,200	-67,512	48	17
PFCIRM134_00130	**1729**	**CyDye**	2-dehydropantoate 2-reductase	Metabolism of coenzymes and prosthetic groups	**panE**	CYTO	32,200	-5,860	8	2
PFCIRM516_03005	**1732**	**CyDye**	L-lactate dehydrogenase	Main glycolytic pathways	**ldh2**	CYTO	34,000	-32,193	27	8
PFCIRM138_02650	**1732**	**CyDye**	L-lactate dehydrogenase	Main glycolytic pathways	**ldh2**	CYTO	33,900	-27,178	19	6
PFCIRM456_03765	**1749**	**CyDye**	Carboxylic ester hydrolase	Metabolism of lipids	**pf667**	CYTO	34,100	-23,663	30	6
PFCIRM516_12170	**1749**	**CyDye**	Carboxylic ester hydrolase	Metabolism of lipids	**pf667**	CYTO	34,100	-9,208	11	3
PFCIRM456_05745	**1775**	**CyDye**	2-deoxy-D-gluconate 3-dehydrogenase		**kduD**	CYTO	43,300	-21,857	16	4

Locus Tag	Cluster	Method	Description	Function	Gene name	SurfG+ Predicted Localization	Molecular Weight (Da)	log(E value)	Coverage (%)	Number Of Unique Peptides
PFCIRM139_06215	**1792**	**Shaving**	Vitamin B12-dependent ribonucleotide reductase	Metabolism of nucleotides and nucleic acids	**nrdJ**	CYTO	104,700	-13,347	5	3
PFCIRM122_05720	**1796**	**CyDye**	Heat shock protein 20 1 (20 kDa chaperone 1)	Protein folding	**hsp20 1**	CYTO	16,900	-37,325	55	7
PFCIRM527_11760	**1796**	**CyDye**	Heat shock protein 20 1 (20 kDa chaperone 1)	Protein folding	**hsp20 1**	CYTO	16,900	-59,483	70	8
PFCIRM139_07475	**1796**	**Shaving**	Heat shock protein 20 1 (20 kDa chaperone 1)	Protein folding	**hsp20 1**	CYTO	16,900	-10,900	25	2
PFCIRM1_12075	**1796**	**Shaving**	Heat shock protein 20 1 (20 kDa chaperone 1)	Protein folding	**hsp20 1**	CYTO	17,200	-19,260	25	2
PFCIRM456_05685	**1815**	**Shaving**	Hypothetical protein	Pouf, without similarity to other proteins		CYTO	7,300	-7,578	38	2
PFCIRM516_10780	**1815**	**Shaving**	Hypothetical protein	Pouf, without similarity to other proteins		CYTO	7,300	-13,112	53	3
PFCIRM1_00200	**1833**	**Shaving**	Cell envelope-related function transcriptional attenuator common d	Transcription regulation	**cpsA1**	PSE	58,700	-6,130	5	2
PFCIRM134_08195	**1866**	**Shaving**	D-lactate dehydrogenase	Metabolism of carbohydrates And related molecules	**dld**	CYTO	63,600	-15,106	13	5
PFCIRM134_09495	**1869**	**Shaving**	Trypsin-like serine protease	Transport/binding of proteins/peptides	**htrA3**	PSE	46,100	-11,154	7	2
PFCIRM456_11855	**1869**	**Shaving**	Trypsin-like serine protease	Transport/binding of proteins/peptides	**htrA3**	PSE	46,300	-19,534	8	2
PFCIRM516_11160	**1869**	**Shaving**	Trypsin-like serine protease	Transport/binding of proteins/peptides	**htrA3**	PSE	46,100	-23,395	16	4
PFCIRM1_00590	**1869**	**Shaving**	Trypsin-like serine protease	Transport/binding of proteins/peptides	**htrA3**	PSE	45,900	-20,022	12	3
PFCIRM118_05525	**1871**	**Shaving**	Penicillin-binding protein (Transglycosylase/transpeptidase)	Cell wall	**ponA**	SECRETED	77,800	-57,144	19	10
PFCIRM121_04405	**1871**	**Shaving**	Penicillin-binding protein (Transglycosylase/transpeptidase)	Cell wall	**ponA**	SECRETED	77,800	-90,147	41	16
PFCIRM122_09570	**1871**	**Shaving**	Penicillin-binding protein (Transglycosylase/transpeptidase)	Cell wall	**ponA**	SECRETED	77,800	-68,602	33	15
PFCIRM129_10570	**1871**	**Shaving**	Penicillin-binding protein (Transglycosylase/transpeptidase)	Cell wall	**ponA**	SECRETED	77,800	-64,186	28	14
PFCIRM134_11530	**1871**	**Shaving**	Penicillin-binding protein (Transglycosylase/transpeptidase)	Cell wall	**ponA**	SECRETED	77,800	-49,125	25	11
PFCIRM139_00390	**1871**	**Shaving**	Penicillin-binding protein (Transglycosylase/transpeptidase)	Cell wall	**ponA**	SECRETED	77,800	-72,676	34	15
PFCIRM456_04145	**1871**	**Shaving**	Penicillin-binding protein (Transglycosylase/transpeptidase)	Cell wall	**ponA**	SECRETED	77,800	-113,950	42	22
PFCIRM514_07250	**1871**	**Shaving**	Penicillin-binding protein (Transglycosylase/transpeptidase)	Cell wall	**ponA**	SECRETED	77,800	-52,983	25	10
PFCIRM516_10390	**1871**	**Shaving**	Penicillin-binding protein (Transglycosylase/transpeptidase)	Cell wall	**ponA**	SECRETED	77,800	-87,235	33	19
PFCIRM527_04365	**1871**	**Shaving**	Penicillin-binding protein (Transglycosylase/transpeptidase)	Cell wall	**ponA**	SECRETED	77,900	-76,983	36	18
PFCIRM1_00605	**1871**	**Shaving**	Penicillin-binding protein (Transglycosylase/transpeptidase)	Cell wall	**ponA**	SECRETED	77,800	-138,177	43	27
PFCIRM134_02140	**1900**	**CyDye**	Oxidoreductase	Miscellaneous		CYTO	23,300	-24,646	41	6
PFCIRM122_00205	**1901**	**CyDye**	Transcriptional regulator, TetR family	Transcription regulation	**tetR1**	CYTO	25,400	-13,614	10	2
PFCIRM139_07635	**1902**	**Shaving**	drug exporters of the RND superfamily	Adaptation to atypical conditions		PSE	87,500	-59,222	16	9
PFCIRM514_07875	**1949**	**CyDye**	Dihydroorotate dehydrogenase	Metabolism of nucleotides and nucleic acids		CYTO	40,600	-60,958	53	15
PFCIRM118_10645	**1971**	**CyDye**	Adenylosuccinate synthetase (IMP--aspartate ligase)	Metabolism of nucleotides and nucleic acids	**purA**	CYTO	46,400	-33,595	25	8
PFCIRM527_09405	**1971**	**CyDye**	Adenylosuccinate synthetase (IMP--aspartate ligase)	Metabolism of nucleotides and nucleic acids	**purA**	CYTO	46,400	-7,585	9	3
PFCIRM118_00325	**2015**	**CyDye**	Aspartyl/glutamyl-tRNA(Asn/Gln) Amidotransferase subunit B	Protein synthesis	**gatB**	CYTO	54,700	-12,246	13	4

Locus Tag	Cluster	Method	Description	Function	Gene name	SurfG+ Predicted Localization	Molecular Weight (Da)	log(E value)	Coverage (%)	Number Of Unique Peptides
PFCIRM122_06340	**2027**	**CyDye**	60 kDa chaperonin 2 (Protein Cpn60 2) (groEL protein 2)	Protein folding	**groL2**	CYTO	56,400	-81,577	41	17
PFCIRM134_04720	**2027**	**CyDye**	60 kDa chaperonin 2 (Protein Cpn60 2) (groEL protein 2)	Protein folding	**groL2**	CYTO	56,400	-88,691	44	19
PFCIRM139_11320	**2027**	**CyDye**	60 kDa chaperonin 2 (Protein Cpn60 2) (groEL protein 2)	Protein folding	**groL2**	CYTO	56,400	-67,173	40	15
PFCIRM1_01975	**2027**	**CyDye**	60 kDa chaperonin 2 (Protein Cpn60 2) (groEL protein 2)	Protein folding	**groL2**	CYTO	56,400	-76,222	33	12
PFCIRM138_06020	**2027**	**CyDye**	60 kDa chaperonin 2 (Protein Cpn60 2) (groEL protein 2)	Protein folding	**groL2**	CYTO	56,400	-124,389	45	19
PFCIRM527_09805	**2027**	**CyDye**	60 kDa chaperonin 2 (Protein Cpn60 2) (groEL protein 2)	Protein folding	**groL2**	CYTO	56,400	-121,820	46	21
PFCIRM134_04720	**2027**	**ClGua**	60 kDa chaperonin 2 (Protein Cpn60 2) (groEL protein 2)	Protein folding	**groL2**	CYTO	56,400	-49,222	24	9
PFCIRM118_10235	**2027**	**Shaving**	60 kDa chaperonin 2 (Protein Cpn60 2) (groEL protein 2)	Protein folding	**groL2**	CYTO	56,400	-38,316	21	8
PFCIRM129_10100	**2027**	**Shaving**	60 kDa chaperonin 2 (Protein Cpn60 2) (groEL protein 2)	Protein folding	**groL2**	CYTO	56,400	-11,724	5	2
PFCIRM134_04720	**2027**	**Shaving**	60 kDa chaperonin 2 (Protein Cpn60 2) (groEL protein 2)	Protein folding	**groL2**	CYTO	56,400	-32,423	23	8
PFCIRM139_11320	**2027**	**Shaving**	60 kDa chaperonin 2 (Protein Cpn60 2) (groEL protein 2)	Protein folding	**groL2**	CYTO	56,400	-25,832	17	6
PFCIRM516_10965	**2027**	**Shaving**	60 kDa chaperonin 2 (Protein Cpn60 2) (groEL protein 2)	Protein folding	**groL2**	CYTO	56,400	-14,845	9	2
PFCIRM1_01975	**2027**	**Shaving**	60 kDa chaperonin 2 (Protein Cpn60 2) (groEL protein 2)	Protein folding	**groL2**	CYTO	56,400	-26,791	18	6
PFCIRM516_00220	**2036**	**CyDye**	Fructose-bisphosphate aldolase class I	Main glycolytic pathways	**fba2**	CYTO	32,200	-35,952	42	9
PFCIRM1_02085	**2036**	**CyDye**	Fructose-bisphosphate aldolase class I	Main glycolytic pathways	**fba2**	CYTO	32,200	-38,417	50	9
PFCIRM122_12120	**2057**	**CyDye**	Glycerol kinase (ATP:glycerol 3-phosphotransferase) (Glycerokinase) (GK)	Specific carbohydrate metabolic pathway	**glpK**	CYTO	55,600	-24,957	15	5
PFCIRM456_08580	**2057**	**CyDye**	Glycerol kinase (ATP:glycerol 3-phosphotransferase) (Glycerokinase) (GK)	Specific carbohydrate metabolic pathway	**glpK**	CYTO	55,600	-21,041	13	5
PFCIRM527_01085	**2057**	**CyDye**	Glycerol kinase (ATP:glycerol 3-phosphotransferase) (Glycerokinase) (GK)	Specific carbohydrate metabolic pathway	**glpK**	CYTO	55,600	-54,770	31	12
PFCIRM118_07490	**2072**	**CyDye**	Methylmalonyl-CoA mutase large subunit	Specific carbohydrate metabolic pathway	**mutB**	CYTO	80,000	-105,297	38	19
PFCIRM118_07490	**2072**	**Shaving**	Methylmalonyl-CoA mutase large subunit	Specific carbohydrate metabolic pathway	**mutB**	CYTO	80,000	-19,964	8	4
PFCIRM122_05020	**2072**	**Shaving**	Methylmalonyl-CoA mutase large subunit	Specific carbohydrate metabolic pathway	**mutB**	CYTO	80,000	-7,152	4	2
PFCIRM129_07240	**2072**	**Shaving**	Methylmalonyl-CoA mutase large subunit	Specific carbohydrate metabolic pathway	**mutB**	CYTO	80,100	-7,877	3	2
PFCIRM134_06200	**2072**	**Shaving**	Methylmalonyl-CoA mutase large subunit	Specific carbohydrate metabolic pathway	**mutB**	CYTO	80,000	-17,165	7	3
PFCIRM122_05025	**2073**	**CyDye**	Methylmalonyl-CoA mutase small subunit	Specific carbohydrate metabolic pathway	**mutA**	CYTO	69,300	-34,339	20	12
PFCIRM129_07235	**2073**	**CyDye**	Methylmalonyl-CoA mutase small subunit	Specific carbohydrate metabolic pathway	**mutA**	CYTO	69,500	-80,319	40	20
PFCIRM118_07495	**2073**	**Shaving**	Methylmalonyl-CoA mutase small subunit	Specific carbohydrate metabolic pathway	**mutA**	CYTO	69,300	-10,203	7	3
PFCIRM129_07235	**2073**	**Shaving**	Methylmalonyl-CoA mutase small subunit	Specific carbohydrate metabolic pathway	**mutA**	CYTO	69,500	-12,045	6	3
PFCIRM134_06205	**2073**	**Shaving**	Methylmalonyl-CoA mutase small subunit	Specific carbohydrate metabolic pathway	**mutA**	CYTO	69,300	-4,687	4	2
PFCIRM134_04125	**2098**	**CyDye**	Glutamine synthetase	Metabolism of nitrogen/nitrate and nitrite	**glnA1**	CYTO	54,000	-18,498	11	4
PFCIRM118_04390	**2098**	**CyDye**	Glutamine synthetase	Metabolism of nitrogen/nitrate and nitrite	**glnA1**	CYTO	54,000	-7,497	5	2
PFCIRM138_02025	**2098**	**CyDye**	Glutamine synthetase	Metabolism of nitrogen/nitrate and nitrite	**glnA1**	CYTO	54,000	-89,215	39	16

Locus Tag	Cluster	Method	Description	Function	Gene name	SurfG+ Predicted Localization	Molecular Weight (Da)	log(E value)	Coverage (%)	Number Of Unique Peptides
PFCIRM121_06890	**2106**	**Shaving**	Cell envelope-related transcriptional attenuator	Transcription regulation		PSE	90,300	-6,858	4	2
PFCIRM456_01865	**2106**	**Shaving**	Cell envelope-related transcriptional attenuator	Transcription regulation		PSE	89,700	-9,076	2	2
PFCIRM514_07715	**2106**	**Shaving**	Cell envelope-related transcriptional attenuator	Transcription regulation		PSE	89,700	-12,994	5	3
PFCIRM516_03570	**2106**	**Shaving**	Cell envelope-related transcriptional attenuator	Transcription regulation		PSE	89,800	-6,890	4	2
PFCIRM1_02665	**2106**	**Shaving**	Cell envelope-related transcriptional attenuator	Transcription regulation		PSE	89,700	-12,101	7	3
PFCIRM516_01560	**2152**	**CyDye**	Dihydrodipicolinate synthase	Metabolism of amino acids and related molecules	**dapA**	CYTO	30,700	-22,487	23	4
PFCIRM527_08455	**2192**	**CyDye**	Electron transfer flavoprotein-quinone oxidoreductase (FixC protein)	Membrane bioenergetics	**fixC (ydiS)**	CYTO	46,400	-43,842	25	7
PFCIRM122_11245	**2275**	**CyDye**	Aspartate ammonia-lyase	Metabolism of amino acids and related molecules	**aspA2**	CYTO	52,900	-40,896	31	12
PFCIRM516_04135	**2491**	**CyDye**	Chaperone clpB 1 (ATP-dependent Clp protease B1) (Clp chaperone)	Protein folding	**clpB 1**	CYTO	94,300	-151,878	49	37
PFCIRM516_09600	**2659**	**Shaving**	Putative uncharacterized protein	Pouf		PSE	28,200	-10,498	10	3
PFCIRM134_01465	**2670**	**Shaving**	protein of unknown function	Pouf, without similarity to other proteins		PSE	36,600	-10,728	9	2
PFCIRM118_01990	**2973**	**CyDye**	Alpha-glucan phosphorylase	Metabolism of carbohydrates and related molecules	**glgP**	CYTO	94,600	-7,000	3	2
PFCIRM118_00365	**3222**	**Shaving**	Beta-lactamase	Detoxification		CYTO	18,800	-14,106	7	2
PFCIRM121_03325	**3222**	**Shaving**	Beta-lactamase	Detoxification		CYTO	20,300	-24,832	37	5
PFCIRM134_04450	**3222**	**Shaving**	Beta-lactamase	Detoxification		CYTO	20,300	-8,606	7	2
PFCIRM514_11870	**3222**	**Shaving**	Beta-lactamase	Detoxification		CYTO	18,800	-37,812	34	6
PFCIRM516_11255	**3222**	**Shaving**	Beta-lactamase	Detoxification		CYTO	20,300	-36,365	28	4
PFCIRM121_07195	**3270**	**Shaving**	Serine protease	Protein modification	**htrA3**	SECRETED	28,200	-12,450	13	2
PFCIRM139_07420	**3303**	**Shaving**	Hypothetical protein	Pouf similar to proteins from other organisms		CYTO	16,900	-5,565	7	2
PFCIRM456_03435	**3303**	**Shaving**	Hypothetical protein	Pouf similar to proteins from other organisms		CYTO	18,700	-6,769	12	2
PFCIRM514_11495	**3303**	**Shaving**	Hypothetical protein	Pouf similar to proteins from other organisms		CYTO	18,700	-23,980	38	6
PFCIRM516_04745	**3303**	**Shaving**	Hypothetical protein	Pouf similar to proteins from other organisms		CYTO	20,200	-13,812	29	3
PFCIRM514_02045	**3384**	**Shaving**	Surface layer protein D (S-layer protein D)	Cell wall		PSE	23,400	-4,052	12	2
PFCIRM516_01510	**3384**	**Shaving**	Surface layer protein D (S-layer protein D)	Cell wall		PSE	23,300	-6,101	14	2
PFCIRM118_04375	**3396**	**Shaving**	Dihydrolipoamide acyltransferase, E2 component of pyruvate dehydrogenase complex	Metabolism of carbohydrates and related molecules	**pdhB**	CYTO	59,500	-18,724	3	2
PFCIRM129_12235	**3671**	**CyDye**	Putative minor extracellular serine protease	Cell wall		PSE	145,500	-14,114	2	2
PFCIRM118_06410	**3671**	**ClGua**	Putative minor extracellular serine protease	Cell wall		PSE	149,600	-161,828	29	25
PFCIRM122_05375	**3671**	**ClGua**	Putative minor extracellular serine protease	Cell wall		PSE	149,600	-201,445	37	35
PFCIRM129_12235	**3671**	**ClGua**	Putative minor extracellular serine protease	Cell wall		PSE	145,500	-186,148	35	32
PFCIRM134_02360	**3671**	**ClGua**	Putative minor extracellular serine protease	Cell wall		PSE	145,500	-166,301	36	31
PFCIRM118_06410	**3671**	**Shaving**	Putative minor extracellular serine protease	Cell wall		PSE	149,600	-150,421	31	28
PFCIRM122_05375	**3671**	**Shaving**	Putative minor extracellular serine protease	Cell wall		PSE	149,600	-351,997	57	61
PFCIRM129_12235	**3671**	**Shaving**	Putative minor extracellular serine protease	Cell wall		PSE	145,500	-290,700	51	54
PFCIRM134_02360	**3671**	**Shaving**	Putative minor extracellular serine protease	Cell wall		PSE	145,500	-357,444	58	67

Locus Tag	Cluster	Method	Description	Function	Gene name	SurfG+ Predicted Localization	Molecular Weight (Da)	log(E value)	Coverage (%)	Number Of Unique Peptides
PFCIRM129_05625	**3715**	**Shaving**	tRNA nucleotidyltransferase PcnB	RNA modification	**pcnB**	CYTO	36,000	-9,333	9	2
PFCIRM134_02110	**3715**	**Shaving**	tRNA nucleotidyltransferase PcnB	RNA modification	**pcnB**	CYTO	36,000	-12,792	23	5
PFCIRM139_07670	**3715**	**Shaving**	tRNA nucleotidyltransferase PcnB	RNA modification	**pcnB**	CYTO	36,000	-18,578	22	5
PFCIRM456_06470	**3715**	**Shaving**	tRNA nucleotidyltransferase PcnB	RNA modification	**pcnB**	CYTO	37,600	-38,010	33	7
PFCIRM514_09515	**3715**	**Shaving**	tRNA nucleotidyltransferase PcnB	RNA modification	**pcnB**	CYTO	37,600	-23,276	25	6
PFCIRM516_00160	**3715**	**Shaving**	tRNA nucleotidyltransferase PcnB	RNA modification	**pcnB**	CYTO	37,600	-15,114	17	4
PFCIRM118_05310	**3762**	**CyDye**	Chaperone clpB 2 (ATP-dependent Clp protease B2) (Clp chaperone)	Protein folding	**clpB 2**	CYTO	94,200	-139,775	37	28
PFCIRM118_05310	**3762**	**Shaving**	Chaperone clpB 2 (ATP-dependent Clp protease B2) (Clp chaperone)	Protein folding	**clpB 2**	CYTO	94,200	-33,590	11	8
PFCIRM129_06355	**3762**	**Shaving**	Chaperone clpB 2 (ATP-dependent Clp protease B2) (Clp chaperone)	Protein folding	**clpB 2**	CYTO	94,200	-7,560	3	2
PFCIRM139_01270	**3762**	**Shaving**	Chaperone clpB 2 (ATP-dependent Clp protease B2) (Clp chaperone)	Protein folding	**clpB 2**	CYTO	94,200	-12,370	4	3
PFCIRM139_01295	**3767**	**Shaving**	Trypsin-like serine protease		**htrA1**	PSE	35,900	-10,434	7	2
PFCIRM118_06850	**3980**	**Shaving**	Phosphoribosyltransferase/nitroreductase (fusion gene)	Metabolism of coenzymes and prosthetic groups	**bluB/cobT**	CYTO	66,300	-10,040	4	2
PFCIRM516_06285	**5421**	**Shaving**	Hypothetical protein	Pouf, without similarity to other proteins		PSE	17,900	-14,724	23	3
PFCIRM1_03640	**5421**	**Shaving**	Hypothetical protein	Pouf, without similarity to other proteins		PSE	18,200	-17,298	22	2
PFCIRM134_10560	**7120**	**Shaving**	secreted protein of unknown function	Pouf similar to proteins from other organisms		PSE	17,600	-10,481	17	3
PFCIRM514_09845	**7120**	**Shaving**	secreted protein of unknown function	Pouf similar to proteins from other organisms		PSE	17,600	-8,014	20	2
PFCIRM516_06675	**8306**	**Shaving**	Hypothetical transmembrane protein	Pouf similar to proteins from other organisms		PSE	75,000	-7,218	2	2
PFCIRM516_11635	**9372**	**CyDye**	L-Lactate dehydrogenase	Main glycolytic pathways	**ldh1**	CYTO	96,900	-34,801	10	6
PFCIRM1_08705	**9372**	**CyDye**	L-Lactate dehydrogenase	Main glycolytic pathways	**alaS**	CYTO	96,900	-43,980	19	11
PFCIRM118_09005	**9372**	**CyDye**	Alanyl-tRNA synthetase (Alanine--tRNA ligase)	Aminoacyl-tRNA synthetases	**alaS**	CYTO	96,900	-39,090	11	7
PFCIRM456_02790	**9429**	**Shaving**	Protein of unknown function	Pouf similar to proteins from other organisms		PSE	17,800	-48,914	33	12
PFCIRM516_11920	**9429**	**Shaving**	Protein of unknown function	Pouf similar to proteins from other organisms		PSE	17,800	-27,646	31	9
PFCIRM139_00285	**10195**	**CyDye**	large surface protein A	Cell wall	**lspA**	SECRETED	115,100	-6,843	4	3
PFCIRM456_04910	**10195**	**CyDye**	large surface protein A	Cell wall	**lspA**	SECRETED	115,200	-10,983	4	4
PFCIRM516_06620	**10195**	**CyDye**	large surface protein A	Cell wall	**lspA**	SECRETED	109,800	-62,858	18	15
PFCIRM139_00285	**10195**	**ClGua**	large surface protein A	Cell wall	**lspA**	SECRETED	115,100	-40,628	11	8
PFCIRM456_04910	**10195**	**ClGua**	large surface protein A	Cell wall	**lspA**	SECRETED	115,200	-41,427	11	9
PFCIRM516_06620	**10195**	**ClGua**	large surface protein A	Cell wall	**lspA**	SECRETED	109,800	-66,200	14	13
PFCIRM139_00285	**10195**	**Shaving**	large surface protein A	Cell wall	**lspA**	SECRETED	115,100	-173,273	37	29
PFCIRM456_04910	**10195**	**Shaving**	large surface protein A	Cell wall	**lspA**	SECRETED	115,200	-260,481	57	46
PFCIRM516_06620	**10195**	**Shaving**	large surface protein A	Cell wall	**lspA**	SECRETED	109,800	-108,848	32	21
PFCIRM121_09990	**12131**	**Shaving**	Leucine Rich Repeat domain protein			SECRETED	113,700	-49,415	22	14
PFCIRM527_06820	**12131**	**Shaving**	Leucine Rich Repeat domain protein			SECRETED	113,700	-59,646	20	13
PFCIRM527_04630	**13599**	**Shaving**	Putative uncharacterized protein			CYTO	14,900	-29,389	43	5
PFCIRM118_01980	**14274**	**Shaving**	Periplasmic binding protein			SECRETED	36,400	-12,126	13	2
PFCIRM134_09660	**14274**	**Shaving**	Periplasmic binding protein			SECRETED	36,300	-75,013	46	12

Locus Tag	Cluster	Method	Description	Function	Gene name	SurfG+ Predicted Localization	Molecular Weight (Da)	log(E value)	Coverage (%)	Number Of Unique Peptides
PFCIRM122_04010	**14403**	**CyDye**	surface layer protein B (S-layer protein B)	Cell wall	**slpB**	PSE	56,500	-19,426	21	9
PFCIRM129_00700	**14403**	**CyDye**	surface layer protein B (S-layer protein B)	Cell wall	**slpB**	PSE	56,800	-46,061	27	14
PFCIRM118_02660	**14403**	**CyDye**	surface layer protein B (S-layer protein B)	Cell wall	**slpB**	PSE	56,500	-19,294	15	5
PFCIRM118_02660	**14403**	**ClGua**	surface layer protein B (S-layer protein B)	Cell wall	**slpB**	PSE	56,500	-81,925	34	13
PFCIRM122_04010	**14403**	**ClGua**	surface layer protein B (S-layer protein B)	Cell wall	**slpB**	PSE	56,500	-23,222	20	7
PFCIRM129_00700	**14403**	**ClGua**	surface layer protein B (S-layer protein B)	Cell wall	**slpB**	PSE	56,800	-78,076	40	18
PFCIRM118_02660	**14403**	**Shaving**	surface layer protein B (S-layer protein B)	Cell wall	**slpB**	PSE	56,500	-178,095	41	32
PFCIRM122_04010	**14403**	**Shaving**	surface layer protein B (S-layer protein B)	Cell wall	**slpB**	PSE	NA	-92,900	NA	23
PFCIRM129_00700	**14403**	**Shaving**	surface layer protein B (S-layer protein B)	Cell wall	**slpB**	PSE	56,800	-189,520	42	33
PFCIRM122_04005	**14404**	**Shaving**	Protein of unknown function	Pouf, without similarity to other proteins		PSE	36,100	-11,028	7	2
PFCIRM129_00705	**14404**	**Shaving**	Protein of unknown function	Pouf, without similarity to other proteins		PSE	36,600	-9,523	8	2
PFCIRM134_00845	**14532**	**CyDye**	Transcriptional regulatory protein	Transcription regulation	**glnR**	CYTO	NA	-52,024	NA	12
PFCIRM139_02295	**14532**	**CyDye**	Transcriptional regulatory protein	Transcription regulation	**glnR**	CYTO	39,100	-19,075	20	5
PFCIRM456_06975	**14532**	**CyDye**	Transcriptional regulatory protein	Transcription regulation	**glnR**	CYTO	39,100	-18,026	31	5
PFCIRM514_02665	**14532**	**CyDye**	Transcriptional regulatory protein	Transcription regulation	**glnR**	CYTO	39,100	-57,960	43	12
PFCIRM118_03315	**14532**	**CyDye**	inositol-1-phosphate synthase	Specific carbohydrate metabolic pathway		CYTO	39,100	-61,313	45	11
PFCIRM527_04635	**14532**	**CyDye**	inositol-1-phosphate synthase	Specific carbohydrate metabolic pathway		CYTO	39,100	-55,437	40	11
PFCIRM118_03315	**14532**	**Shaving**	Transcriptional regulatory protein	Transcription regulation	**glnR**	CYTO	39,100	-19,205	16	4
PFCIRM118_03320	**14532**	**Shaving**	Transcriptional regulatory protein	Transcription regulation	**glnR**	CYTO	18,300	-19,981	27	3
PFCIRM121_02375	**14532**	**Shaving**	Transcriptional regulatory protein	Transcription regulation	**glnR**	CYTO	18,300	-50,239	49	7
PFCIRM134_00845	**14532**	**Shaving**	Transcriptional regulatory protein	Transcription regulation	**glnR**	CYTO	39,100	-10,237	8	2
PFCIRM139_02295	**14532**	**Shaving**	Transcriptional regulatory protein	Transcription regulation	**glnR**	CYTO	39,100	-17,482	12	3
PFCIRM456_06975	**14532**	**Shaving**	Transcriptional regulatory protein	Transcription regulation	**glnR**	CYTO	39,100	-13,565	8	2
PFCIRM139_00025	**17478**	**Shaving**	Protein of unknown function	Pouf, without similarity to other proteins		CYTO	30,700	-10,699	20	2
PFCIRM139_02065	**17495**	**Shaving**	Protein of unknown function	Pouf, without similarity to other proteins		CYTO	35,300	-5,680	8	2
PFCIRM121_05625	**19508**	**ClGua**	large surface protein A	Cell wall	**lspA**	SECRETED	137,100	-37,437	9	8
PFCIRM121_05625	**19508**	**Shaving**	large surface protein A	Cell wall	**lspA**	SECRETED	137,100	-134,232	24	27
PFCIRM139_01875	**44324**	**Shaving**	Putative uncharacterized protein			SECRETED	15,300	-31,154	56	7
PFCIRM139_01925	**44334**	**Shaving**	Putative uncharacterized protein			CYTO	29,600	-14,677	19	3
PFCIRM456_06970	**47711**	**Shaving**	Protein of unknown function	Pouf similar to proteins from other organisms		CYTO	16,100	-46,790	56	7

Locus Tag	Cluster	Method	Description	Function	Gene name	SurfG+ Predicted Localization	Molecular Weight (Da)	log(E value)	Coverage (%)	Number Of Unique Peptides
PFCIRM138_06935	**ND**	**CyDye**	ND		**ND**	ND	19,100	-14,201	28	4
PFCIRM138_07450	**ND**	**CyDye**	ND		**ND**	ND	34,100	-10,985	7	2
PFCIRM122_11065	**ND**	**CyDye**	ND		**ND**	ND	NA	-20,250	NA	7
PFCIRM122_04025	**ND**	**CyDye**	ND	Adaptation to atypical conditions	**ND**	ND	NA	-20,250	NA	7
PFCIRM134_05115	**ND**	**CyDye**	ND		**ND**	ND	NA	-56,055	NA	15
PFCIRM134_08980	**ND**	**CyDye**	ND		**ND**	ND	NA	-56,055	NA	15
PFCIRM134_05120	**ND**	**CyDye**	ND		**ND**	ND	NA	-56,055	NA	15
PFCIRM134_08985	**ND**	**CyDye**	ND		**ND**	ND	NA	-58,238	NA	15
PFCIRM139_10980	**ND**	**CyDye**	ND		**ND**	ND	NA	-25,500	NA	8
PFCIRM129_03685	**ND**	**ClGua**	ND		**ND**	ND	NA	-16,409	NA	3
PFCIRM129_03680	**ND**	**ClGua**	ND		**ND**	ND	NA	-16,400	NA	3
PFCIRM134_01655	**ND**	**ClGua**	ND		**ND**	ND	NA	-24,126	NA	5
PFCIRM134_12765	**ND**	**ClGua**	ND		**ND**	ND	NA	-24,126	NA	5
PFCIRM138_06935	**ND**	**ClGua**	ND		**ND**	ND	NA	-35,455	NA	7
PFCIRM514_09110	**ND**	**ClGua**	ND		**ND**	ND	NA	-44,732	NA	10
PFCIRM514_09085	**ND**	**ClGua**	ND		**ND**	ND	NA	-44,732	NA	10
PFCIRM118_01020	**ND**	**Shaving**	ND		**ND**	ND	NA	-15,930	NA	3
PFCIRM118_01025	**ND**	**Shaving**	ND		**ND**	ND	NA	-15,930	NA	3
PFCIRM122_11065	**ND**	**Shaving**	ND		**ND**	ND	NA	-51,700	NA	12
PFCIRM122_04025	**ND**	**Shaving**	ND		**rimM**	ND	NA	-51,700	NA	12
PFCIRM129_03680	**ND**	**Shaving**	ND		**ND**	ND	NA	-111,600	NA	16
PFCIRM129_03685	**ND**	**Shaving**	ND		**ND**	ND	NA	-111,600	NA	16
PFCIRM129_05040	**ND**	**Shaving**	ND		**ND**	ND	NA	-10,200	NA	2
PFCIRM134_01655	**ND**	**Shaving**	ND		**ND**	ND	NA	-69,100	NA	13
PFCIRM134_12765	**ND**	**Shaving**	ND		**ND**	ND	NA	-69,100	NA	13
PFCIRM134_07870	**ND**	**Shaving**	ND		**ND**	ND	NA	-11,400	NA	3
PFCIRM134_07875	**ND**	**Shaving**	ND		**ND**	ND	NA	-11,400	NA	3
PFCIRM138_06935	**ND**	**Shaving**	ND		**ND**	ND	NA	-4,300	NA	2
PFCIRM138_07450	**ND**	**Shaving**	ND		**ND**	ND	NA	-4,300	NA	2
PFCIRM139_08365	**ND**	**Shaving**	ND		**ND**	ND	NA	-66,700	NA	13
PFCIRM514_09085	**ND**	**Shaving**	ND		**ND**	ND	NA	-243,800	NA	48
PFCIRM514_09110	**ND**	**Shaving**	ND		**ND**	ND	NA	-243,800	NA	48
PFCIRM527_10380	**ND**	**Shaving**	ND		**ND**	ND	NA	-62,900	NA	14
PFCIRM527_05765	**ND**	**Shaving**	ND		**ND**	ND	NA	-62,900	NA	14
